# Supplementary material for: Exosomes harbor B cell targets in pancreatic adenocarcinoma and exert decoy function against complement-mediated cytotoxicity
Source: Nat Commun. 2019 Jan 16;10:254. doi: 10.1038/s41467-018-08109-6 (PMC6335434; doi:10.1038/s41467-018-08109-6)
Supplement: Supplementary file 1 — Supplementary Information [file 41467_2018_8109_MOESM1_ESM.pdf]

# Supplementary Information

## Exosomes harbor B cell targets in pancreatic adenocarcinoma and exert decoy function against complement-mediated cytotoxicity

Michela Capello, Jody V. Vykoukal, Hiroyuki Katayama,
Leonidas E. Bantis, Hong Wang, Deepali L. Kundnani,
Clemente Aguilar-Bonavides, Mitzi Aguilar, Satyendra C. Tripathi,
Dilsher S. Dhillon, Amin A. Momin, Haley Peters, Matthew H. Katz,
Hector Alvarez, Vincent Bernard, Sammy Ferri-Borgogno, Randall Brand,
Douglas G. Adler, Matthew A. Firpo, Sean J. Mulvihill,
Jeffrey J. Molldrem, Ziding Feng, Ayumu Taguchi, Anirban Maitra and
Samir M. Hanash

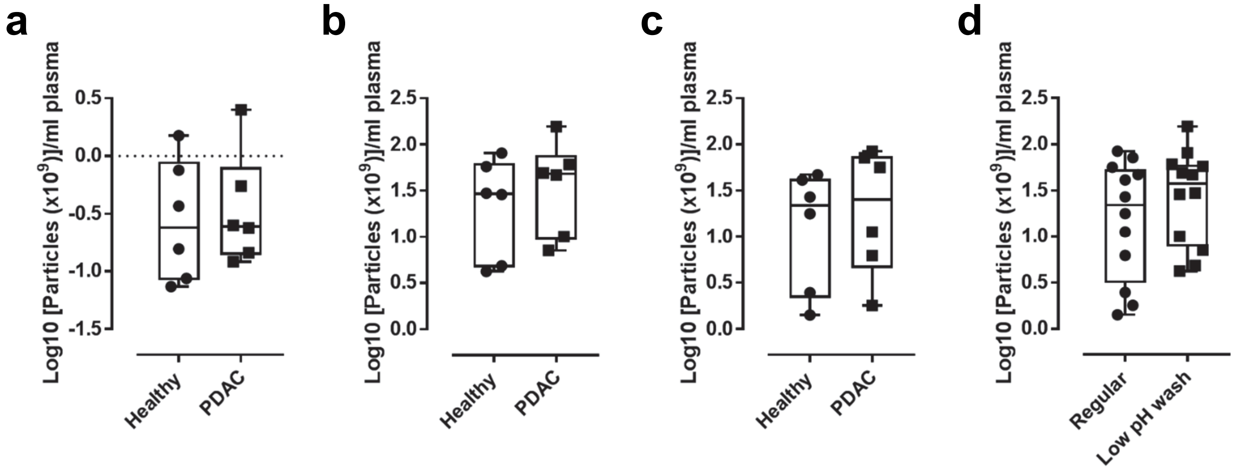


**Supplementary Figure 1.** Testing of the non-specific binding of exosomes to Nab Protein A/G columns using PDAC and healthy control plasma samples (*n* = 6 per group). Quantification by nanoparticle-tracking of exosome (30-200 nm size) counts in: (**a**) the flow-through of a low pH wash (pH 5) performed following plasma sample loading; (**b**) the Ig bound elute following a low pH wash (pH 5); and (**c**) the Ig bound elute without performing a low pH wash. (**d**) Graph depicting no significant change in the number of exosomes in the Ig elute performed without (regular) or following (low pH wash) a low pH wash. Boxes indicate 25th and 75th percentiles, and horizontal lines inside the boxes indicate median. Bars indicate maximum and minimum.

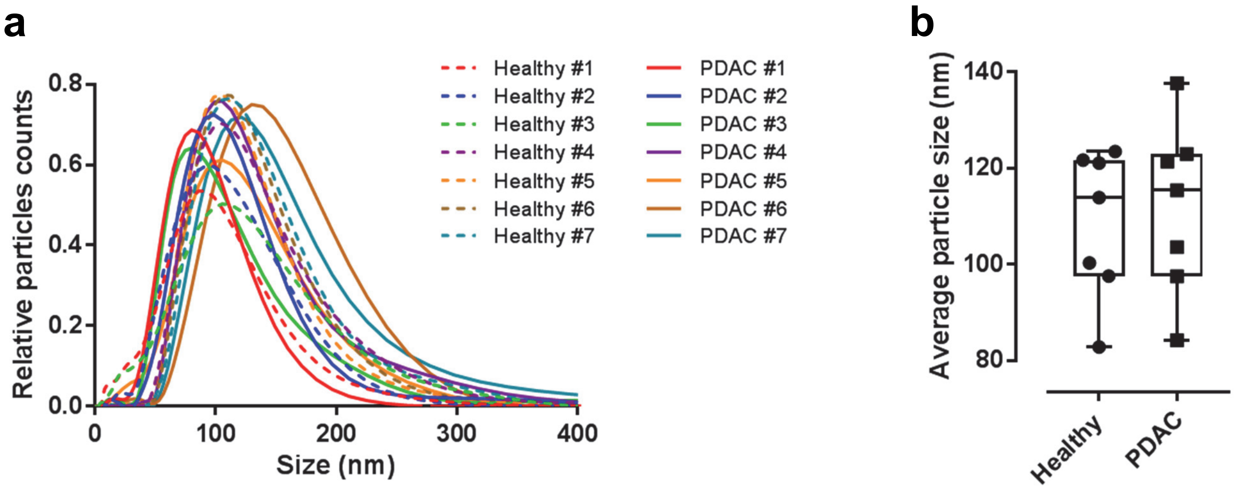


**Supplementary Figure 2.** Isolation and characterization of exosomes bound to circulating Igs in the plasma of PDAC patients and healthy controls (*n* = 7 per group). (**a**) Nanoparticle-tracking analysis of exosomes isolated from the plasma Ig-bound fraction of seven PDAC patients and seven matched healthy controls indicating particle size distribution of individual samples. (**b**) Graph represents the average size of particles quantified in the Ig-bound fraction of PDAC patients and matched healthy controls by nanoparticle-tracking analysis. Boxes indicate 25th and 75th percentiles, and horizontal lines inside the boxes indicate median. Bars indicate maximum and minimum.

**a**

| | | Pancreatic cancer | Healthy controls |
|---|---|---|---|
| Total (n) | | 6 | 6 |
| Gender (n) | Male | 4 | 4 |
| | Female | 2 | 2 |
| Age (mean (range)) | | 65 (55 - 76) | 60 (50 -69) |
| Stage (n) | IV | 6 | - |

**b**

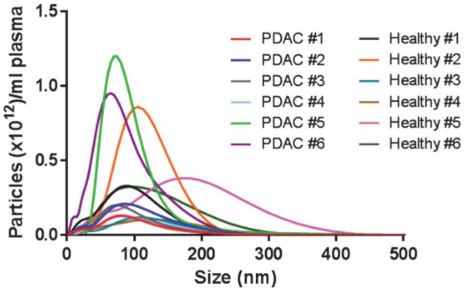


| | Particle size (nm) | | Particle count (x10$^{12}$)* | |
|---|---|---|---|---|
| | Mean | SD | Mean | SD |
| PDAC | 74.2 | 7.7 | 7.2 | 6.7 |
| Healthy | 97.8 | 18.4 | 5.8 | 3.5 |

*Number of vesicles with a 30-200 nm size

**c**

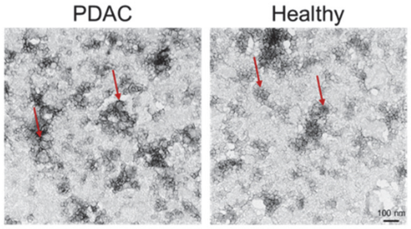


**d**

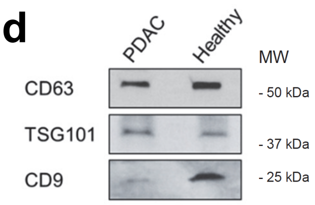


**Supplementary Figure 3.** Isolation and characterization of exosomes from PDAC patient plasma. (**a**) Patient characteristics of PDAC and healthy subject plasma samples applied for extracellular vesicles isolation and mass spectrometry analysis. (**b**) Nanoparticle-tracking analysis of exosomes isolated from the plasma of six PDAC patients and six matched healthy controls indicating size distribution of isolated particles. (**c**) Representative TEM micrograph of exosomes isolated from the plasma of a PDAC patient and a healthy subject. Arrows indicate vesicles with classical exosome size and morphology. (**d**) Representative Western-blot analysis of exosome markers CD63, TSG101, and CD9 expression in plasma exosomes from a PDAC patient and a healthy subject.

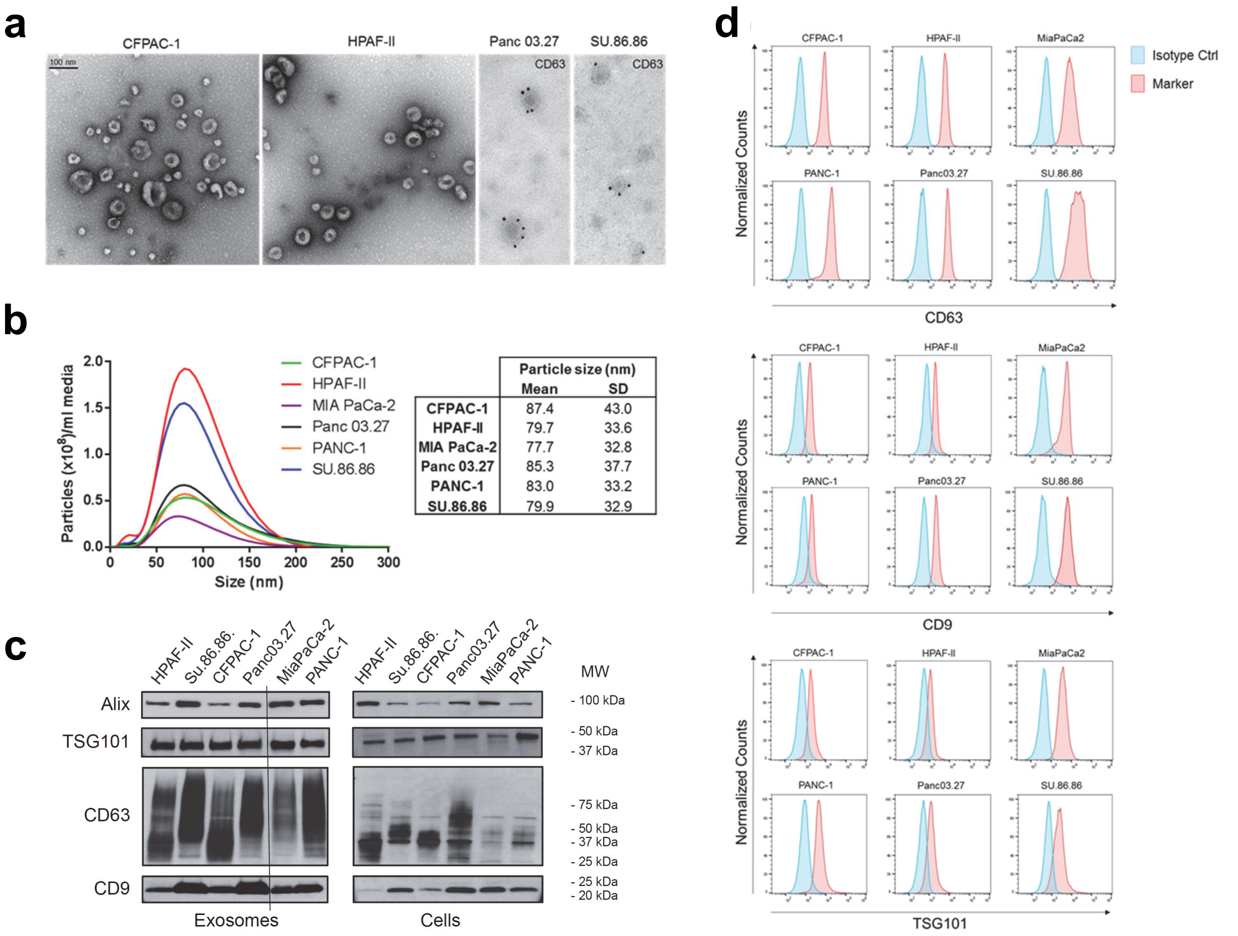


**Supplementary Figure 4.** Characterization of exosomes isolated from PDAC cell line conditioned media. (**a**) Representative TEM micrograph of CFPAC-1 and HPAF-II cell line exosomes (left images). Representative TEM micrograph of the immunogold labeling of Panc 03.27 and SU.86.86 cell line exosomes using anti-CD63 antibody (right images). Black dots indicate gold particles. (**b**) Nanoparticle-tracking analysis of exosomes isolated from the panel of six PDAC cell lines, indicating size distribution of isolated particles. (**c**) Western-blot analysis of exosome markers Alix, TSG101, CD63 and CD9 expression in total and exosome PDAC cell line protein extracts. (**d**) Flow cytometry analysis using exosome markers CD63, CD9, and TSG101 of PDAC cell line exosomes coupled to 0.4 µm-diameter beads.

**a**

| KEGG Pathway Name | -Log(Adj p-value) | Size | Ratio |
|---|---|---|---|
| Cell adhesion molecules (CAMs) | 20.8 | 25 | 0.188 |
| Phagosome | 17.0 | 23 | 0.150 |
| Proteasome | 13.5 | 13 | 0.295 |
| Spliceosome | 11.8 | 17 | 0.134 |
| Antigen processing and presentation | 11.7 | 14 | 0.184 |
| Protein processing in endoplasmic reticulum | 10.2 | 17 | 0.103 |
| Regulation of actin cytoskeleton | 9.4 | 18 | 0.085 |
| Focal adhesion | 8.0 | 16 | 0.080 |
| Systemic lupus erythematosus | 7.4 | 13 | 0.096 |
| Allograft rejection | 7.4 | 8 | 0.216 |
| Ribosome | 7.4 | 11 | 0.120 |
| ECM-receptor interaction | 6.7 | 10 | 0.118 |
| Natural killer cell mediated cytotoxicity | 6.7 | 12 | 0.088 |
| Endocytosis | 6.5 | 14 | 0.070 |
| Fc gamma R-mediated phagocytosis | 6.4 | 10 | 0.106 |
| Autoimmune thyroid disease | 6.4 | 8 | 0.154 |
| Lysosome | 6.4 | 11 | 0.091 |
| SNARE interactions in vesicular transport | 6.4 | 7 | 0.194 |
| Axon guidance | 6.1 | 11 | 0.085 |
| Graft-versus-host disease | 6.0 | 7 | 0.171 |
| Type I diabetes mellitus | 5.7 | 7 | 0.163 |

| Ingenuity Canonical Pathways | -Log(Adj p-value) | Size | Ratio |
|---|---|---|---|
| Protein Ubiquitination Pathway | 10.5 | 27 | 0.106 |
| Caveolar-mediated Endocytosis Signaling | 10.3 | 15 | 0.211 |
| Antigen Presentation Pathway | 9.3 | 11 | 0.289 |
| Phagosome Maturation | 9.15 | 19 | 0.132 |
| Virus Entry via Endocytic Pathways | 8.92 | 16 | 0.157 |
| Axonal Guidance Signaling | 7.32 | 31 | 0.069 |
| Cdc42 Signaling | 7.3 | 18 | 0.108 |
| Ephrin Receptor Signaling | 7.02 | 18 | 0.103 |
| Clathrin-mediated Endocytosis Signaling | 6.89 | 19 | 0.096 |
| Regulation of Actin-based Motility by Rho | 6.86 | 13 | 0.143 |
| Ephrin B Signaling | 6.14 | 11 | 0.151 |
| Tight Junction Signaling | 5.86 | 16 | 0.096 |
| RhoGDI Signaling | 5.66 | 16 | 0.093 |
| Integrin Signaling | 4.94 | 17 | 0.078 |
| Actin Cytoskeleton Signaling | 4.72 | 17 | 0.075 |
| EIF2 Signaling | 4.32 | 16 | 0.072 |
| Systemic Lupus Erythematosus Signaling | 4.23 | 16 | 0.071 |
| Autoimmune Thyroid Disease Signaling | 4.07 | 7 | 0.149 |
| Leukocyte Extravasation Signaling | 4.02 | 15 | 0.071 |
| Allograft Rejection Signaling | 3.92 | 9 | 0.107 |
| Crosstalk between Dendritic Cells and Natural Killer Cells | 3.73 | 9 | 0.101 |

**b**

| Gene Symbol | Gene Name | Entrez Gene ID | Pathway Analysis |
|---|---|---|---|
| CD40 | CD40 molecule, TNF receptor superfamily member 5 | 958 | KEGG; IPA |
| H2AFJ | H2A histone family, member J | 55766 | KEGG |
| H2AFV | H2A histone family, member V | 94239 | KEGG |
| H2AFX | H2A histone family, member X | 3014 | KEGG |
| H3F3C | H3 histone, family 3C | 440093 | KEGG |
| HIST1H2AJ | histone cluster 1, H2aj | 8331 | KEGG |
| HIST2H2BE | histone cluster 2, H2be | 8349 | KEGG |
| HIST2H3A | histone cluster 2, H3a | 333932 | KEGG |
| HIST3H2BB | histone cluster 3, H2bb | 128312 | KEGG |
| HLA-A | major histocompatibility complex, class I, A | 3105 | IPA |
| HLA-B | major histocompatibility complex, class I, B | 3106 | IPA |
| HLA-C | major histocompatibility complex, class I, C | 3107 | IPA |
| HLA-DPB1 | major histocompatibility complex, class II, DP beta 1 | 3115 | KEGG |
| HLA-DRB1 | major histocompatibility complex, class II, DR beta 1 | 3123 | KEGG |
| HLA-E | major histocompatibility complex, class I, E | 3133 | IPA |
| HLA-G | major histocompatibility complex, class I, G | 3135 | IPA |
| HNRNPA2B1 | heterogeneous nuclear ribonucleoprotein A2/B1 | 3181 | IPA |
| HNRNPC | heterogeneous nuclear ribonucleoprotein C (C1/C2) | 3183 | IPA |
| SF3B4 | splicing factor 3b subunit 4 | 10262 | IPA |
| SNRPA | small nuclear ribonucleoprotein polypeptide A | 6626 | IPA |
| SNRPD1 | small nuclear ribonucleoprotein D1 polypeptide | 6632 | KEGG; IPA |
| SNRPD2 | small nuclear ribonucleoprotein D2 polypeptide | 6633 | IPA |
| SNRPD3 | small nuclear ribonucleoprotein D3 polypeptide | 6634 | KEGG; IPA |
| SNRPF | small nuclear ribonucleoprotein polypeptide F | 6636 | IPA |
| SNRPG | small nuclear ribonucleoprotein polypeptide G | 6637 | IPA |
| SNU13 | SNU13 homolog, small nuclear ribonucleoprotein (U4/U6.U5) | 4809 | IPA |

**c**

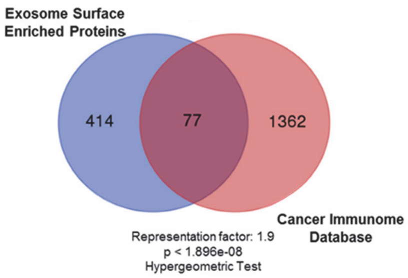


**Supplementary Figure 5.** Identification of PDAC exosome surface enrichment of antigens able to induce autoantibodies. (**a**) Top canonical pathways predicted by KEGG and IPA pathway analyses for 491 proteins enriched on the exosome surface compared to total lysate and cargo compartment (**Figure 4c**). Size indicates the number of genes in the input list belonging to each specific pathway. Ratio indicates the ratio between the number of entities in the pathway and the number of genes in the input list belonging to that pathway. Immune-related pathways are underlined. (**b**) List of proteins enriched on the exosome surface belonging to the systemic lupus erythematosus pathway. (**c**) Venn diagram showing overlap of exosome surface enriched proteins and the Cancer Immunome Database. *p*-value was calculated by hypergeometric test.

**a**

| KEGG Pathway Name | -Log(Adj p-value) | Size | Ratio |
|---|---|---|---|
| Proteasome | 19.0 | 15 | 0.341 |
| Phagosome | 11.8 | 16 | 0.105 |
| Endocytosis | 11.2 | 17 | 0.085 |
| Ribosome | 7.5 | 10 | 0.109 |
| RNA transport | 7.5 | 12 | 0.079 |
| Vasopressin-regulated water reabsorption | 6.3 | 7 | 0.159 |
| Spliceosome | 6.3 | 10 | 0.079 |
| Cell adhesion molecules (CAMs) | 6.2 | 10 | 0.075 |
| Systemic lupus erythematosus | 6.2 | 10 | 0.074 |
| SNARE interactions in vesicular transport | 5.7 | 6 | 0.167 |
| Lysosome | 5.7 | 9 | 0.074 |
| Allograft rejection | 5.7 | 6 | 0.162 |
| Gap junction | 5.7 | 8 | 0.089 |
| Graft-versus-host disease | 5.5 | 6 | 0.146 |
| Tight junction | 5.5 | 9 | 0.068 |
| Type I diabetes mellitus | 5.4 | 6 | 0.140 |
| Antigen processing and presentation | 5.1 | 7 | 0.092 |
| Autoimmune thyroid disease | 5.0 | 6 | 0.115 |
| Pancreatic secretion | 4.4 | 7 | 0.069 |
| Salivary secretion | 3.7 | 6 | 0.067 |
| Natural killer cell mediated cytotoxicity | 3.5 | 7 | 0.051 |
| Leukocyte transendothelial migration | 3.2 | 6 | 0.052 |
| Protein processing in endoplasmic reticulum | 3.2 | 7 | 0.042 |

| Ingenuity Canonical Pathways | -Log(Adj p-value) | Size | Ratio |
|---|---|---|---|
| Protein Ubiquitination Pathway | 12.2 | 24 | 0.094 |
| Phagosome Maturation | 9.4 | 16 | 0.111 |
| EIF2 Signaling | 8.3 | 18 | 0.081 |
| Clathrin-mediated Endocytosis Signaling | 6.63 | 15 | 0.076 |
| Tight Junction Signaling | 5.93 | 13 | 0.078 |
| Cdc42 Signaling | 5.16 | 12 | 0.072 |
| RhoGDI Signaling | 5 | 12 | 0.069 |
| CTLA4 Signaling in Cytotoxic T Lymphocytes | 4.82 | 12 | 0.091 |
| Antigen Presentation Pathway | 4.75 | 9 | 0.158 |
| mTOR Signaling | 4.4 | 6 | 0.060 |
| Epithelial Adherens Junction Signaling | 4.21 | 12 | 0.069 |
| Autoimmune Thyroid Disease Signaling | 4.21 | 10 | 0.128 |
| Remodeling of Epithelial Adherens Junctions | 4.21 | 7 | 0.103 |
| Graft-versus-Host Disease Signaling | 4.16 | 6 | 0.125 |
| Caveolar-mediated Endocytosis Signaling | 4.09 | 7 | 0.099 |
| Ephrin B Signaling | 4.01 | 7 | 0.096 |
| Virus Entry via Endocytic Pathways | 3.88 | 8 | 0.078 |
| Axonal Guidance Signaling | 3.42 | 17 | 0.038 |
| ILK Signaling | 3.18 | 10 | 0.051 |
| CXCR4 Signaling | 3.12 | 9 | 0.055 |
| Role of NFAT in Regulation of the Immune Response | 2.77 | 9 | 0.049 |
| Systemic Lupus Erythematosus Signaling | 2.73 | 10 | 0.044 |
| Crosstalk between Dendritic Cells and Natural Killer Cells | 2.7 | 6 | 0.067 |

**b**

| Gene Symbol | Gene Name | Entrez Gene ID | Pathway Analysis |
|---|---|---|---|
| H2AFJ | H2A histone family, member J | 55766 | KEGG |
| H2AFV | H2A histone family, member V | 94239 | KEGG |
| H2AFX | H2A histone family, member X | 3014 | KEGG |
| H3F3C | H3 histone, family 3C | 440093 | KEGG |
| HIST1H2AJ | histone cluster 1, H2aj | 8331 | KEGG |
| HIST2H2BE | histone cluster 2, H2be | 8349 | KEGG |
| HIST2H3A | histone cluster 2, H3a | 333932 | KEGG |
| HIST3H2BB | histone cluster 3, H2bb | 128312 | KEGG |
| HLA-A | major histocompatibility complex, class I, A | 3105 | IPA |
| HLA-B | major histocompatibility complex, class I, B | 3106 | IPA |
| HLA-C | major histocompatibility complex, class I, C | 3107 | IPA |
| HLA-DRB1 | major histocompatibility complex, class II, DR beta 1 | 3123 | KEGG |
| HLA-E | major histocompatibility complex, class I, E | 3133 | IPA |
| HLA-G | major histocompatibility complex, class I, G | 3135 | IPA |
| HNRNPC | heterogeneous nuclear ribonucleoprotein C (C1/C2) | 3183 | IPA |
| NHP2L1 | SNU13 homolog, small nuclear ribonucleoprotein (U4/U6.U5) | 4809 | IPA |
| SNRPA | small nuclear ribonucleoprotein polypeptide A | 6626 | IPA |
| SNRPD3 | small nuclear ribonucleoprotein D3 polypeptide | 6634 | IPA; KEGG |
| SNRPF | small nuclear ribonucleoprotein polypeptide F | 6636 | IPA |

**c**

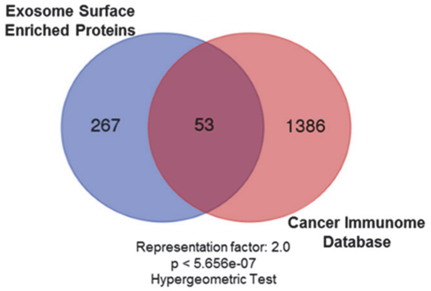


**Supplementary Figure 6.** Identification of PDAC exosome surface enrichment in antigens compared to cell surface. (**a**) Top canonical pathways predicted by KEGG and IPA pathway analyses for the cluster of proteins enriched on the exosome surface compared to cell surface (**Figure 4c**). Size indicates the number of genes in the input list belonging to each specific pathway. Ratio indicate the ratio between the number of entities in the pathway and the number of genes in the input list belonging to that pathway. Immune-related pathways are underlined. (**b**) List of proteins enriched on the exosome surface compared to cell surface belonging to the systemic lupus erythematosus pathway. (**c**) Venn diagram showing overlap of exosome surface enriched proteins and the Cancer Immunome Database. *p*-value was calculated by hypergeometric test.

- Exosome Surface (9/361) vs Total Exosome Extract (2/454) Enrichment: P=0.0141
- Exosome Surface Identified (at least 10 MS2; 24/483) vs Exosome Surface Not Identified (10/1130): P<0.0001
- Exosome Surface (18/316) vs Cell Surface (4/563) Enrichment : P<0.0001
- Total Exosome Extract (16/306) vs Total Cell Extract (3/503) Enrichment: P<0.0001

Fisher's exact test

**Supplementary Figure 7.** Enrichment analysis of PDAC Ig-bound protein expression in PDAC cells and exosomes. Analysis refers to **Figure 5a** and Supplementary **Table 5** and enrichment was defined as follow, Surface-to-TEE enrichment: proteins with surface-to-TEE MS2 count ratio ≥2; Surface identified: proteins identified on the surface with normalized MS2 counts ≥10; Exosome surface-to-cell surface enrichment: proteins significantly (*p*-value<0.05; Mann-Whitney t-test) higher on exosome surface compared to cell surface; TEE-to-TCE enrichment: proteins significantly (*p*-value<0.05; Mann-Whitney t-test) higher in TEE compared to TCE. Numbers indicate the ratio between the number of proteins identified in the Ig-bound fraction and the total number of identified proteins for that specific proteome. *p*-values were calculated by Fisher's exact test. TCE, total cell extract; TEE, total exosome extract.

| Gene | HLA-II | Immunoblot | Plasma Exosomes | Protein Array |
|---|---|---|---|---|
| ACTA1 | | X | | |
| ***ACTB*** | X | X | X | |
| ANXA1 | | X | | |
| ARF6 | | | | |
| ATP5B | | | | |
| GSTP1 | X | | | |
| ***H2A variants*** | | X | X | |
| ***HSPA8*** | X | X | X | |
| JUP | | | X | |
| KRT10 | X | | | |
| KRT16 | | | X | |
| KRT17 | | | X | |
| ***KRT20*** | X | | | |
| ***KRT5*** | X | | X | |
| ***LGALS3BP*** | | | X | X |
| NAGK | | | | |
| NME2P1 | | | | |
| PABPC4 | | | | |
| PCBP1 | X | | | |
| PDE4DIP | | | | |
| ***PKM/PKM2*** | X | X | X | X |
| POTEE | | | | |
| POTEF | | | | |
| PSMA7 | | X | | |
| RAN | | X | | |
| RPSA | | X | | |
| ***TUBB*** | X | X | | |
| TUBB2A | | | | |
| TUBB2B | | X | | |
| TUBB3 | | X | | |
| TUBB4A | | X | | |
| TUBB4B | | X | | |
| UBA52 | X | | | |
| UBC | | | | |

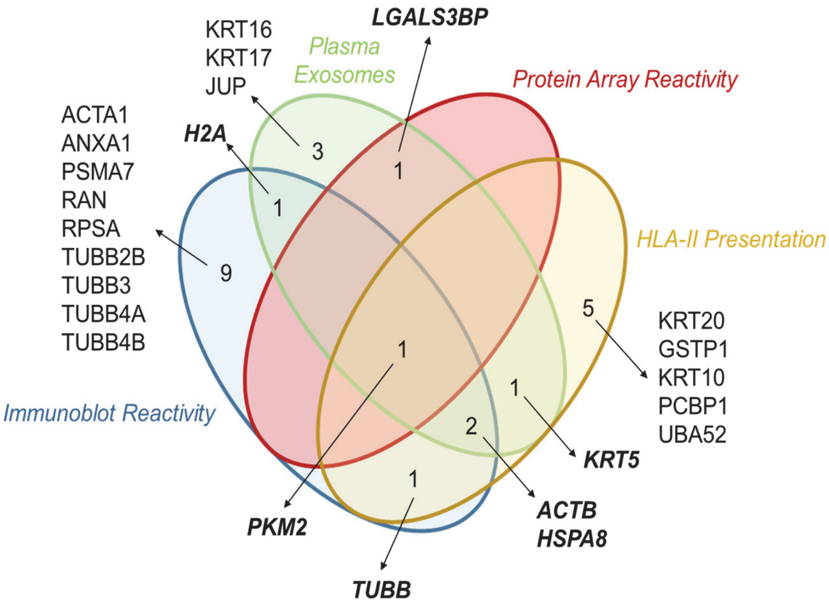


**Supplementary Figure 8.** The intersected proteomic profiling of PDAC antigens identified in patient Ig-bound fraction, cell line- and plasma-derived exosome, HLA-II immunopeptidome, immunoblot and protein array autoantibody analysis. Table and Venn diagram showing overlap of proteins present in the PDAC plasma Ig-bound fraction and in cell lines exosomes also identified in additional analyses. Highlighted proteins were identified in at least two independent analyses.

**a**

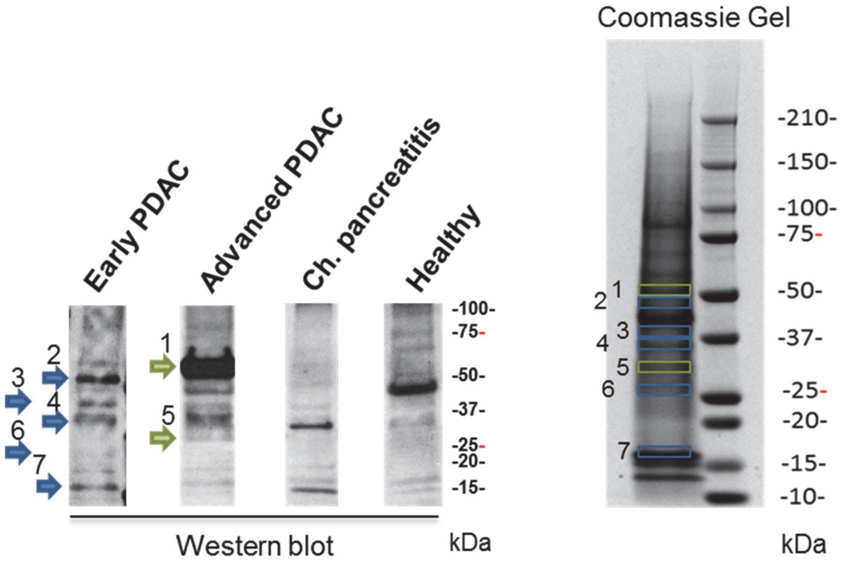


**b**

| | Surface | | | | Surface Enrichment Ratio | | Total Extract | | | |
|---|---|---|---|---|---|---|---|---|---|---|
| Gene | Cell Mean MS2 | Exosome Mean MS2 | Ratio Exosome/Cell | P Value | Cells | Exosomes | Cell Mean MS2 | Exosome Mean MS2 | Ratio Exosome/Cell | P Value |
| ACTA1 | 2.8 | 358.5 | 129.57 | **0.0379** | 2.8 | 0.85 | NA | 421.8 | 421.78 | **0.0027** |
| ACTB | 193.9 | 266.9 | 1.38 | 0.2403 | 0.8 | 0.84 | 254.1 | 317.3 | 1.25 | 0.3768 |
| ANXA1 | 187.6 | 107.3 | 0.57 | 0.0411 | 0.7 | 1.55 | 265.9 | 69.2 | 0.26 | 0.0152 |
| H2AFV/X | NA | 141.3 | 141.27 | **0.0028** | 0.0 | 7.36 | NA | 19.2 | 19.20 | **0.0028** |
| HSPA8 | NA | 749.6 | 749.55 | **0.0027** | 0.0 | 1.03 | NA | 726.3 | 726.34 | **0.0027** |
| PKM/PKM2 | 637.9 | 629.2 | 0.99 | 1.0000 | 1.0 | 0.60 | 582.4 | 1053.2 | 1.81 | **0.0260** |
| PSMA7 | 1.4 | 201.5 | 142.62 | **0.0037** | 0.1 | 4.75 | 20.1 | 42.4 | 2.10 | 0.0776 |
| RAN | 3.4 | 46.9 | 13.93 | **0.0278** | 3.4 | 0.46 | NA | 101.1 | 101.10 | **0.0028** |
| RPSA | 4.5 | 72.7 | 16.05 | **0.0022** | 0.1 | 1.21 | 36.0 | 60.0 | 1.67 | **0.0152** |
| TUBB | 102.2 | 581.1 | 5.68 | **0.0050** | 1.4 | 1.00 | 75.0 | 578.8 | 7.72 | **0.0050** |
| TUBB2B | NA | 148.2 | 148.21 | **0.0028** | 0.0 | 0.93 | NA | 159.8 | 159.79 | **0.0028** |
| TUBB3 | 2.3 | 308.5 | 136.25 | **0.0022** | 0.9 | 0.99 | 2.6 | 312.3 | 121.17 | **0.0050** |
| TUBB4A | 1.9 | 269.0 | 145.26 | **0.0050** | 1.7 | 1.15 | 1.1 | 233.2 | 217.33 | **0.0037** |
| TUBB4B | 23.3 | 178.0 | 7.64 | **0.0022** | 1.0 | 1.01 | 23.6 | 176.0 | 7.46 | **0.0022** |

**Supplementary Figure 9**. (**a**) Western blot analysis of PDAC patient plasma reactivity against cell line exosome protein lysate. Protein lysates from the panel of 6 PDAC cell lines were combined, separated by SDS-PAGE gels and subsequently immunoblotted with plasma pools (left panel) or Coomassie stained (right panel). Western blot images show the immunoreactivity of plasma from patients with early-stage PDAC, advanced PDAC, matched healthy subjects and chronic pancreatitis controls. Immunoreactive protein spots were determined by superimposition of immunoblot signal pattern with the Coomassie stained gel. Numbered arrows and squares indicate immunoreactive proteins specifically recognized by PDAC patient plasma and identified by LC-MS/MS. (**b**) Table showing the average spectral abundance (MS2 counts) in total lysate and surface proteome of PDAC cells and exosomes of antigens identified in PDAC plasma Ig-bound fraction, cell lines exosomes and immunoblot analysis of PDAC patient plasma reactivity. Surface enrichment indicates the ratio of surface to total extract MS2 counts. *p*-value was calculated by Mann-Whitney t-test.

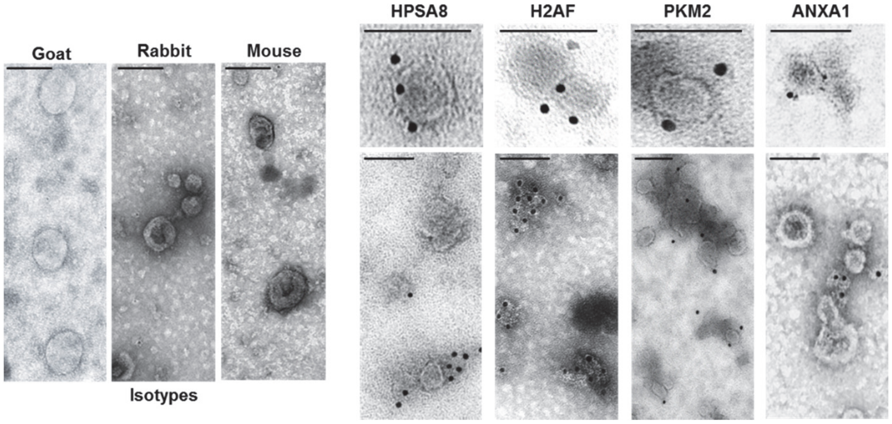


**Supplementary Figure 10.** Localization analysis of antigens able to induce autoantibodies in PDAC patients. Representative TEM micrograph of the immunogold labeling of exosomes isolated from PDAC cell lines using anti-HSPA8, H2AF, PKM2 and ANXA1 or isotype control antibodies. Black dots indicate gold particles. Scale bars indicate 100 nm.

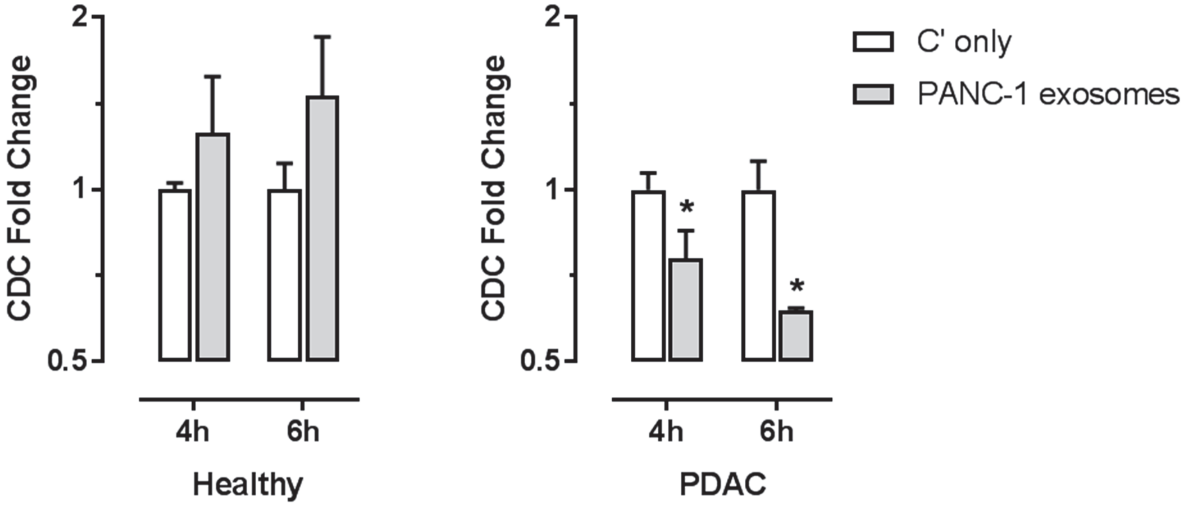


**Supplementary Figure 11.** Complement-dependent cytotoxicity of PANC-1 cells in presence of exosomes, isolated from the same cell line, mediated by pre-diagnostic PDAC sera or matched healthy controls from the CARET study (pool, $n = 13$). Data are expressed as fold change in the number of green dead cells relative to the respective controls (sera plus complement in absence of exosomes; C' only) at two different time points after complement incubation. Graphs illustrate the mean result ± s.d. of triplicates from a representative experiment of three replicates. *p*-values were calculated by two-sided unpaired t-test. *$p$-value $< 0.05$.

**Supplementary Table 1.** Patient characteristics of PDAC plasma sample cohorts applied for analysis of Ig-bound proteins and exosomes.

| | | Cohort #1 | | Cohort #2 | | | Cohort #3 | | Cohort #4 | |
|---|---|---|---|---|---|---|---|---|---|---|
| | | Pancreatic cancer | Pancreatic Cyst | Pancreatic cancer | Healthy controls | Chronic pancreatitis | Pancreatic cancer | Healthy controls | Pancreatic cancer | Healthy controls |
| Total (*n*) | | 28 | 12 | 32 | 32 | 32 | 12 | 12 | 6 | 6 |
| Gender (*n*) | Male | 16 | 2 | 20 | 20 | 20 | 6 | 5 | 4 | 4 |
| | Female | 12 | 10 | 12 | 12 | 12 | 6 | 7 | 2 | 2 |
| Age (mean (s.d.)) | | 65.9 (10.6) | 69.4 (13.2) | 67 (10.3) | 65.8 (9.2) | 60.4 (9.3) | 55.1 (8.1) | 50.1 (10.1) | 65 (55 - 76) | 60 (50 -69) |
| Type-2 diabetes | *Yes* | 11 | 4 | - | - | - | - | - | - | - |
| | *No* | 17 | 8 | - | - | - | - | - | - | - |
| Stage (*n*) | IA | - | - | 3 | - | - | - | - | - | - |
| | IB | - | - | 9 | - | - | - | - | - | - |
| | IIA | - | - | 20 | - | - | - | - | - | - |
| | Potentially Resectable | 18 | - | - | - | - | 18 | - | - | - |
| | Locally Advanced | 10 | - | - | - | - | 10 | - | - | - |
| | Unresectable (No TNM data) | - | - | - | - | - | 12 | - | 6 | - |
| Timing of sample collection | | At time of diagnosis | | At time of diagnosis | | | At time of diagnosis | | At time of diagnosis | |
| **Analysis** | **Figure** | | | | | | | | | |
| *Ig bound proteomics* | *Fig. 1* | X | | X | | | | | | |
| *Exosome in Ig bound fraction* | *Fig. 2, Supp. Fig. 1 & 2* | | | | | | X | | | |
| *Plasma exosome proteomics* | *Fig. 3, Fig. 5d, Supp. Fig. 3* | | | | | | | | X | |

**Supplementary Table 2.** Proteins identified by mass spectrometry analysis in the Ig-bound fraction of PDAC sample Cohort #1 at higher levels in PDAC *vs.* matched controls

| Gene | PDAC *vs* Cyst (MS2 Ratio)* | | | | | TCGA† | PDAC Cell Line RNASeq‡ | | Oncomine Overexpression |
|---|---|---|---|---|---|---|---|---|---|
| | PR w T2D | LA w T2D | PR w/o T2D | LA w/o T2D | *Average* | Mean (RSEM) | Mean (FPKM) | N. Cell line ID& | % Studies# |
| **ABI3BP** | NA | NA | 2.9 | NA | ***2.9*** | 943.60 | 3.55 | 3 | 17 |
| **AIF1L** | NA | NA | NA | 3.7 | ***3.7*** | 269.63 | 11.12 | 10 | 0 |
| **AJUBA** | 4.6 | NA | NA | NA | ***4.6*** | 481.14 | 37.55 | 11 | 67 |
| **APITD1** | 4.6 | NA | NA | NA | ***4.6*** | 218.17 | 6.43 | 10 | NA |
| **ATP5B** | NA | NA | NA | 10.8 | ***10.8*** | 9764.70 | 379.60 | 11 | 33 |
| **ATP9B** | NA | 2.0 | NA | 7.4 | ***4.7*** | 404.70 | 2.87 | 10 | 14 |
| **BBIP1** | NA | NA | NA | 5.4 | ***5.4*** | 238.32 | 2.85 | 9 | 43 |
| **BOP1** | NA | NA | 3.7 | NA | ***3.7*** | 352.47 | 57.36 | 11 | 29 |
| **BPNT1** | 4.6 | NA | NA | NA | ***4.6*** | 860.44 | 14.26 | 11 | 57 |
| **C12orf73** | NA | 4.6 | NA | NA | ***4.6*** | 108.05 | 1.90 | 10 | 50 |
| **C17orf97** | 7.5 | NA | NA | NA | ***7.5*** | 89.05 | 1.84 | 6 | 0 |
| **C3orf67** | NA | 4.6 | NA | NA | ***4.6*** | 37.24 | 1.71 | 7 | 25 |
| **C6orf52** | 4.6 | NA | NA | NA | ***4.6*** | 6.78 | 0.72 | 3 | 0 |
| **CAMK2D** | NA | 3.7 | NA | NA | ***3.7*** | 1194.05 | 4.04 | 10 | 43 |
| **CD79A** | NA | NA | NA | 9.1 | ***9.1*** | 661.74 | 0.80 | 2 | 13 |
| **COL8A2** | NA | NA | NA | 5.4 | ***5.4*** | 1160.21 | 0.48 | 2 | 67 |
| **CREBRF** | 4.6 | NA | NA | NA | ***4.6*** | 412.20 | 2.64 | 9 | 20 |
| **DCP1A** | NA | NA | NA | 3.7 | ***3.7*** | 693.64 | 6.43 | 11 | 57 |
| **ITPR2** | NA | 8.2 | NA | 23.9 | ***16.1*** | 1080.63 | 2.66 | 9 | 25 |
| **JUP** | 1.6 | 0.1 | 6.7 | 10.8 | ***4.8*** | 12206.66 | 84.44 | 11 | 63 |
| **KIAA1467** | 4.6 | NA | NA | NA | ***4.6*** | 164.76 | 2.50 | 11 | 14 |
| **KRT10** | 2.9 | 1.9 | 0.5 | 0.9 | ***1.6*** | 511.25 | 9.17 | 11 | 0 |
| **KRT17** | 2.0 | 0.1 | 17.5 | 23.9 | ***10.9*** | 13407.08 | 236.10 | 7 | 71 |
| **LGALS3BP** | 1.2 | 1.7 | 6.1 | 1.9 | ***2.7*** | 17650.20 | 311.49 | 11 | 75 |
| **LTB4R** | 12.2 | NA | NA | NA | ***12.2*** | 224.03 | 6.34 | 10 | 33 |
| **LUC7L** | NA | NA | NA | 3.7 | ***3.7*** | 855.25 | 14.59 | 11 | 14 |
| **MED24** | 17.1 | NA | NA | 27.4 | ***22.3*** | 1465.53 | 16.99 | 11 | 25 |
| **MFSD7** | NA | NA | NA | 3.7 | ***3.7*** | 263.67 | 1.58 | 4 | 0 |
| **MGA** | NA | NA | 4.6 | NA | ***4.6*** | 801.53 | 4.68 | 11 | 43 |
| **MLF2** | NA | NA | NA | 3.7 | ***3.7*** | 3250.14 | 78.47 | 11 | 38 |
| **NAGK** | NA | NA | NA | 3.7 | ***3.7*** | 1224.52 | 16.10 | 11 | 33 |
| **NF1** | NA | NA | 11.6 | NA | ***11.6*** | 1813.19 | 8.09 | 11 | 38 |
| **OBSL1** | 4.6 | NA | NA | NA | ***4.6*** | 1818.69 | 15.22 | 10 | 14 |
| **PABPC4** | 4.6 | NA | NA | NA | ***4.6*** | 3374.30 | 39.12 | 11 | 0 |
| **PDE4DIP** | NA | 3.7 | NA | NA | ***3.7*** | 1966.15 | 8.71 | 11 | 100 |
| **PIGR** | 11.7 | 4.5 | 4.7 | 2.1 | ***5.8*** | 13363.28 | 3.02 | 4 | 50 |
| **POLR2M** | 4.6 | NA | NA | 3.7 | ***4.2*** | 822.22 | 9.25 | 11 | 29 |
| **POTEE** | 5.5 | 0.5 | NA | 2.4 | ***2.8*** | 5.09 | 2.83 | 11 | NA |
| **PPFIBP1** | 4.6 | NA | NA | NA | ***4.6*** | 1474.72 | 9.22 | 11 | 57 |
| **PRKX** | 9.1 | NA | NA | NA | ***9.1*** | 544.34 | 4.96 | 11 | 25 |
| **PRSS53** | NA | NA | 2.9 | NA | ***2.9*** | 55.34 | 2.74 | 8 | 0 |
| **RAN** | 4.6 | NA | NA | NA | ***4.6*** | 3756.40 | 321.27 | 11 | 43 |
| **RERG** | 4.6 | NA | NA | NA | ***4.6*** | 340.76 | 0.74 | 3 | 17 |
| **SHF** | 4.6 | NA | NA | NA | ***4.6*** | 278.81 | 1.94 | 5 | 50 |
| **SIDT2** | 4.6 | NA | NA | NA | ***4.6*** | 1307.02 | 9.06 | 11 | 0 |
| **SLC22A5** | NA | NA | NA | 5.4 | ***5.4*** | 300.16 | 5.26 | 11 | 17 |
| **SLFN13** | 4.6 | NA | NA | NA | ***4.6*** | 520.32 | 4.04 | 8 | 75 |
| **SLMO1** | 4.6 | NA | NA | NA | ***4.6*** | 41.79 | 1.24 | 6 | 0 |
| **SMDT1** | NA | NA | NA | 3.7 | ***3.7*** | 823.56 | 7.89 | 11 | 0 |
| **TCF20** | NA | NA | NA | 3.7 | ***3.7*** | 1162.82 | 9.12 | 11 | 38 |
| **TEX9** | NA | 3.7 | NA | NA | ***3.7*** | 70.07 | 0.89 | 4 | 60 |
| **TMEM106C** | NA | NA | NA | 3.7 | ***3.7*** | 1100.86 | 23.76 | 11 | 0 |
| **TMEM175** | NA | NA | NA | 3.7 | ***3.7*** | 490.22 | 8.74 | 11 | 17 |
| **TMSB10** | 0.6 | 2.1 | 2.4 | 8.3 | ***3.4*** | 28687.71 | 2089.79 | 11 | 88 |
| **TOB2** | 4.6 | NA | NA | NA | ***4.6*** | 1938.16 | 19.74 | 11 | 25 |
| **TOX4** | NA | NA | NA | 3.7 | ***3.7*** | 1553.33 | 19.41 | 11 | 43 |
| **ZFYVE16** | NA | NA | NA | 3.7 | ***3.7*** | 793.25 | 6.13 | 11 | 43 |
| **ZNF385A** | NA | NA | NA | 3.7 | ***3.7*** | 680.89 | 11.49 | 11 | 50 |
| **ZNF461** | NA | 3.7 | NA | NA | ***3.7*** | 89.09 | 1.60 | 10 | 20 |
| **ZNF559** | NA | NA | NA | 5.4 | ***5.4*** | 230.76 | 1.31 | 7 | 17 |
| **ZNF701** | NA | NA | NA | 3.7 | ***3.7*** | 116.73 | 1.84 | 7 | 33 |

*NA* indicates protein not quantified in either of the compared groups
* MS2 count ratio of PDAC case over matched cyst control pools (Figure 1B). PR, potentially resectable. LA, locally advanced. T2D, type 2 diabetes.
† Gene expression level in The Cancer Genome Atlas (TCGA) PDAC dataset (n=112 patients) .
‡ Gene expression level in the panel of 11 PDAC cell lines
& Number of PDAC cell lines (out of 11) in which the RNA for the protein was quantified
# Percentage of the 8 PDAC datasets present in the Oncomine gene expression database in which the protein was reported as significantly overexpressed in PDAC compared to normal adjacent tissue.

No protein was reported as significantly downregulated

**Supplementary Table 3.** Proteins identified by mass spectrometry analysis in the Ig-bound fraction of PDAC sample Cohort #2 at higher levels in PDAC *vs.* matched controls

***Cohort #2***

| Gene | Stage I PDAC (MS2 Ratio) | | | Stage II PDAC (MS2 Ratio) | | | TCGA† | PDAC Cell Line RNASeq‡ | | Oncomine Overexpression |
|---|---|---|---|---|---|---|---|---|---|---|
| | ***vs* Healthy*** | ***vs* Ch. Pancreatitis**** | ***Average*** | ***vs* Healthy*** | ***vs* Ch. Pancreatitis**** | ***Average*** | **Mean (RSEM)** | **Mean (FPKM)** | **N. Cell line ID &** | **% Studies#** |
| **ACTA1** | 4.1 | 4.1 | ***4.1*** | 0.9 | NA | *0.9* | 6.14 | 1.19 | 9 | 0 |
| **ACTB** | 2.5 | 2.5 | ***2.5*** | 1.9 | 1.8 | ***1.9*** | 117377.96 | 2519.80 | 11 | 67 |
| **ANXA1** | 5.6 | 5.6 | ***5.6*** | 1.5 | 1.7 | ***1.6*** | 7496.13 | 334.33 | 11 | 75 |
| **ARF6** | 2.5 | 2.5 | ***2.5*** | 0.9 | NA | *0.9* | 3520.64 | 53.14 | 11 | 50 |
| **AZGP1** | 0.9 | 0.9 | *0.9* | 5.9 | 1.4 | ***3.7*** | 1720.29 | 5.94 | 6 | 14 |
| **CSTA** | 4.1 | 0.7 | ***2.4*** | 1.9 | 0.5 | *1.2* | 265.44 | 7.83 | 3 | 75 |
| **GSTP1** | 3.3 | 3.3 | ***3.3*** | 0.9 | NA | *0.9* | 11042.89 | 379.13 | 11 | 50 |
| **H2AFV** | 0.7 | 0.9 | *0.8* | 1.9 | 1.0 | ***1.5*** | 2654.77 | 50.60 | 11 | 43 |
| **HSPA8** | 2.1 | 2.1 | ***2.1*** | 0.9 | NA | *0.9* | 18191.88 | 562.42 | 11 | 29 |
| **KIAA1429** | 2.5 | 2.5 | ***2.5*** | 0.2 | 1.0 | *0.6* | 1225.64 | 13.33 | 11 | 33 |
| **KRT16** | 1.6 | 17.0 | ***9.3*** | 1.1 | 0.1 | *0.6* | 2114.33 | 4.69 | 8 | 50 |
| **KRT20** | 2.1 | 2.1 | ***2.1*** | 1.9 | 0.3 | *1.1* | 496.47 | 5.60 | 2 | 25 |
| **KRT5** | 2.0 | 3.0 | ***2.5*** | 0.7 | 0.1 | *0.4* | 2453.15 | 277.53 | 5 | 38 |
| **LGALS3BP** | 1.4 | 1.4 | *1.4* | 4.1 | 2.2 | ***3.2*** | 17650.20 | 311.49 | 11 | 75 |
| **NME2P1** | 2.1 | 2.1 | ***2.1*** | 0.9 | NA | *0.9* | 7901.75 | 297.53 | 11 | NA |
| **PABPC4** | 3.3 | 3.3 | ***3.3*** | 1.5 | 1.7 | ***1.6*** | 3374.30 | 39.12 | 11 | 0 |
| **PCBP1** | 2.5 | 2.5 | ***2.5*** | 0.9 | NA | *0.9* | 7053.22 | 156.09 | 11 | 25 |
| **PKM** | 1.3 | 2.4 | ***1.9*** | 7.6 | 2.5 | ***5.1*** | 31565.48 | 900.02 | 11 | 71 |
| **PKM2** | 2.1 | 2.1 | ***2.1*** | 0.9 | 0.8 | *0.9* | 31565.48 | 900.02 | 11 | 71 |
| **POTEE** | 0.6 | 4.1 | ***2.4*** | 5.0 | 3.4 | ***4.2*** | 5.09 | 2.83 | 11 | NA |
| **POTEF** | 2.1 | 2.1 | ***2.1*** | 2.5 | 1.3 | ***1.9*** | 13.52 | 0.92 | 2 | NA |
| **PSMA7** | 4.1 | 4.1 | ***4.1*** | 0.9 | NA | *0.9* | 3049.80 | 251.12 | 11 | 67 |
| **RPSA** | 2.1 | 2.1 | ***2.1*** | 0.9 | NA | *0.9* | 6901.50 | 242.15 | 11 | 0 |
| **S100A9** | 1.7 | 1.7 | ***1.7*** | 2.5 | 2.8 | ***2.7*** | 929.29 | 37.87 | 4 | 25 |
| **STXBP6** | 2.1 | 2.1 | ***2.1*** | 0.9 | NA | *0.9* | 227.41 | 0.68 | 3 | 0 |
| **TMSB10** | 3.3 | 0.6 | ***2.0*** | 0.8 | 2.5 | ***1.7*** | 28687.71 | 2089.79 | 11 | 88 |
| **TUBB** | 23.5 | 23.5 | ***23.5*** | 0.9 | NA | *0.9* | 13009.67 | 532.32 | 11 | 71 |
| **TUBB2A** | 5.8 | 5.8 | ***5.8*** | 0.9 | NA | *0.9* | 1194.83 | 21.48 | 11 | 29 |
| **TUBB2B** | 6.9 | 6.9 | ***6.9*** | 0.9 | NA | *0.9* | 127.97 | 11.19 | 11 | 60 |
| **TUBB3** | 7.8 | 7.8 | ***7.8*** | 0.9 | NA | *0.9* | 1439.17 | 66.31 | 11 | 43 |
| **TUBB4A** | 3.3 | 3.3 | ***3.3*** | 0.9 | NA | *0.9* | 101.05 | 5.98 | 5 | 0 |
| **TUBB4B** | 5.6 | 5.6 | ***5.6*** | 0.9 | NA | *0.9* | 6026.78 | 292.57 | 11 | 33 |
| **UBA52** | 4.1 | 4.1 | ***4.1*** | 1.9 | 0.9 | *1.4* | 10053.60 | 49.15 | 11 | 0 |
| **UBC** | 2.1 | 2.1 | ***2.1*** | 0.9 | 0.7 | *0.8* | 37498.44 | 652.17 | 11 | 57 |
| **UBE3D** | 0.9 | 0.9 | *0.9* | 3.2 | 3.6 | ***3.4*** | 48.77 | 2.04 | 10 | 33 |

*NA* indicates protein not quantified in either of the compared groups
* MS2 counts ratio of PDAC case over matched healthy control pools (Figure 1B).
** MS2 counts ratio of PDAC case over matched chronic pancreatitis pools (Figure 1B).
† Gene expression level in The Cancer Genome Atlas (TCGA) PDAC dataset (n=112 patients).
‡ Gene expression level in the panel of 11 PDAC cell lines
& Number of PDAC cell lines (out of 11) in which the RNA for the protein was quantified
# Percentage of the 8 PDAC datasets present in the Oncomine gene expression database in which the protein was reported as significantly overexpressed in PDAC compared to normal adjacent tissue.

No protein was reported as significantly downregulated

**Supplementary Table 4.** Proteins involved in exosome biogenesis, vesicular trafficking, and cytoskeletal regulation identified in plasma extracellular vesicles by mass spectrometry analysis

| Gene Name | Protein Description | Relative Ion Intensity Mean | s.d. |
|---|---|---|---|
| **Exosome Biogenesis** | | | |
| STAMBP | STAM-binding protein OS=Homo sapiens GN=STAMBP PE=1 SV=1 - [STABP_HUMAN] | 1.18E+00 | 1.72E+00 |
| VPS25 | Isoform of Q9BRG1, Vacuolar protein-sorting-associated protein 25 OS=Homo sapiens GN=VPS25 PE=1 SV=1 - [K7EP45_HUMAN] | 1.37E+01 | 1.16E+02 |
| CHMP3 | Isoform of Q9Y3E7, Isoform 2 of Charged multivesicular body protein 3 OS=Homo sapiens GN=CHMP3 - [CHMP3_HUMAN] | 8.65E+00 | 1.25E+02 |
| SDC1 | Syndecan-1 OS=Homo sapiens GN=SDC1 PE=1 SV=3 - [SDC1_HUMAN] | 1.08E+00 | 1.31E+00 |
| SMPD2 | Sphingomyelin phosphodiesterase 2 OS=Homo sapiens GN=SMPD2 PE=1 SV=2 - [NSMA_HUMAN] | 2.04E+00 | 1.07E+01 |
| ABCA1 | "Isoform of O95477, ATP-binding cassette sub-family A member 1 OS=Homo sapiens GN=ABCA1 PE=1 SV=1 - [B1AMI2_HUMAN];""ATP-binding cassette sub-family A member 1 OS=Homo sapiens GN=ABCA1 PE=1 SV=3 - [ABCA1_HUMAN]""" | 1.49E+01 | 1.22E+02 |
| CD151 | Isoform of P48509, Tetraspanin (Fragment) OS=Homo sapiens GN=CD151 PE=1 SV=1 - [E9PJC8_HUMAN] | 1.71E+00 | 5.98E+00 |
| **Vesicular Trafficking** | | | |
| RAB11FIP1 | Isoform of Q6WKZ4, Isoform 4 of Rab11 family-interacting protein 1 OS=Homo sapiens GN=RAB11FIP1 - [RFIP1_HUMAN] | 1.08E+00 | 1.31E+00 |
| RAB11FIP4 | Isoform of Q86YS3, Isoform 2 of Rab11 family-interacting protein 4 OS=Homo sapiens GN=RAB11FIP4 - [RFIP4_HUMAN] | 3.38E+00 | 5.70E+01 |
| RAB12 | Ras-related protein Rab-12 OS=Homo sapiens GN=RAB12 PE=1 SV=3 - [RAB12_HUMAN] | 2.61E+00 | 2.41E+01 |
| RAB1B | Isoform of Q9H0U4, Ras-related protein Rab-1B OS=Homo sapiens GN=RAB1B PE=1 SV=1 - [E9PLD0_HUMAN] | 2.67E+00 | 2.59E+01 |
| RAB21 | Ras-related protein Rab-21 OS=Homo sapiens GN=RAB21 PE=1 SV=3 - [RAB21_HUMAN] | 2.42E+00 | 7.05E+00 |
| RAB3GAP1 | Isoform of Q15042, Isoform 3 of Rab3 GTPase-activating protein catalytic subunit OS=Homo sapiens GN=RAB3GAP1 - [RB3GP_HUMAN] | 2.73E+00 | 2.80E+01 |
| RAB3GAP2 | Rab3 GTPase-activating protein non-catalytic subunit OS=Homo sapiens GN=RAB3GAP2 PE=1 SV=1 - [RBGPR_HUMAN] | 1.72E+00 | 6.00E+00 |
| RAB3IL1 | Isoform of Q8TBN0, Guanine nucleotide exchange factor for Rab-3A (Fragment) OS=Homo sapiens GN=RAB3IL1 PE=1 SV=8 - [E9PK89_HUMAN] | 2.88E+00 | 3.34E+01 |
| RAB40A | Ras-related protein Rab-40A OS=Homo sapiens GN=RAB40A PE=2 SV=2 - [RB40A_HUMAN] | 5.61E+00 | 6.15E+01 |
| RAB44 | "Isoform of Q7Z6P3, Ras-related protein Rab-44 OS=Homo sapiens GN=RAB44 PE=1 SV=1 - [A0A087WXI0_HUMAN];""Ras-related protein Rab-44 OS=Homo sapiens GN=RAB44 PE=1 SV=3 - [RAB44_HUMAN]""" | 2.59E+00 | 8.83E+00 |
| RAB5A | Isoform of P20339, Ras-related protein Rab-5A OS=Homo sapiens GN=RAB5A PE=1 SV=1 - [F8WCY6_HUMAN] | 2.40E+00 | 1.83E+01 |
| RAB6C | Protein WTH3DI OS=Homo sapiens GN=RAB6C PE=1 SV=1 - [Q53S08_HUMAN] | 2.65E+00 | 2.54E+01 |
| RABEP2 | Isoform of Q9H5N1, Rab GTPase-binding effector protein 2 OS=Homo sapiens GN=RABEP2 PE=1 SV=1 - [B4DHR0_HUMAN] | 2.64E+00 | 2.49E+01 |
| RABGAP1 | "Isoform of Q9Y3P9, Isoform 3 of Rab GTPase-activating protein 1 OS=Homo sapiens GN=RABGAP1 - [RBGP1_HUMAN];""Isoform of Q9Y3P9, Rab GTPase-activating protein 1 (Fragment) OS=Homo sapiens GN=RABGAP1 PE=1 SV=1 - [B5MCD9_HUMAN]""" | 4.79E+00 | 4.09E+01 |
| RABGEF1 | Isoform of Q9UJ41, Isoform 2 of Rab5 GDP/GTP exchange factor OS=Homo sapiens GN=RABGEF1 - [RABX5_HUMAN] | 2.62E+00 | 2.45E+01 |
| RABGGTA | Isoform of Q92696, Geranylgeranyl transferase type-2 subunit alpha OS=Homo sapiens GN=RABGGTA PE=1 SV=1 - [H0YKP6_HUMAN] | 2.06E+00 | 1.10E+01 |
| RAP1B | Isoform of P61224, Ras-related protein Rap-1b (Fragment) OS=Homo sapiens GN=RAP1B PE=1 SV=1 - [F5H823_HUMAN] | 8.21E+00 | 1.11E+02 |
| RABIF | Guanine nucleotide exchange factor MSS4 OS=Homo sapiens GN=RABIF PE=1 SV=2 - [MSS4_HUMAN] | 2.65E+00 | 2.53E+01 |
| RHOBTB1 | Rho-related BTB domain-containing protein 1 OS=Homo sapiens GN=RHOBTB1 PE=2 SV=2 - [RHBT1_HUMAN] | 1.16E+00 | 1.65E+00 |
| RHOBTB3 | Rho-related BTB domain-containing protein 3 OS=Homo sapiens GN=RHOBTB3 PE=1 SV=2 - [RHBT3_HUMAN] | 1.18E+00 | 1.73E+00 |
| RHOH | Isoform of Q15669, Rho-related GTP-binding protein RhoH (Fragment) OS=Homo sapiens GN=RHOH PE=1 SV=1 - [D6RG23_HUMAN] | 1.09E+00 | 1.31E+00 |

| | | | |
|---|---|---|---|
| RHOV | Rho-related GTP-binding protein RhoV OS=Homo sapiens GN=RHOV PE=1 SV=1 - [RHOV_HUMAN] | 1.17E+00 | 1.70E+00 |
| ATP6V0D2 | Isoform of Q8N8Y2, V-type proton ATPase subunit d 2 (Fragment) OS=Homo sapiens GN=ATP6V0D2 PE=1 SV=1 - [E5RIR3_HUMAN] | 2.72E+00 | 2.77E+01 |
| ATP6V1B2 | V-type proton ATPase subunit B, brain isoform OS=Homo sapiens GN=ATP6V1B2 PE=1 SV=3 - [VATB2_HUMAN] | 1.28E+00 | 2.27E+00 |
| GDI2 | Isoform of P50395, Rab GDP dissociation inhibitor beta (Fragment) OS=Homo sapiens GN=GDI2 PE=1 SV=8 - [V9GYJ7_HUMAN] | 1.89E+00 | 8.31E+00 |
| CLTC | Isoform of Q00610, Isoform 2 of Clathrin heavy chain 1 OS=Homo sapiens GN=CLTC - [CLH1_HUMAN] | 1.75E+00 | 6.34E+00 |
| VAMP2 | Isoform of P63027, Vesicle-associated membrane protein 2 OS=Homo sapiens GN=VAMP2 PE=4 SV=1 - [K7ENK9_HUMAN] | 1.90E+00 | 8.42E+00 |
| VPS51 | Isoform of Q9UID3, Vacuolar protein sorting-associated protein 51 homolog (Fragment) OS=Homo sapiens GN=VPS51 PE=1 SV=1 - [E9PMB6_HUMAN] | 1.77E+00 | 6.68E+00 |
| VPS52 | Isoform of Q8N1B4, Vacuolar protein sorting-associated protein 52 homolog OS=Homo sapiens GN=VPS52 PE=1 SV=1 - [E9PI03_HUMAN] | 1.59E+00 | 4.62E+00 |
| VPS53 | Isoform of Q5VIR6, Vacuolar protein sorting-associated protein 53 homolog (Fragment) OS=Homo sapiens GN=VPS53 PE=1 SV=1 - [I3L184_HUMAN] | 2.62E+00 | 2.44E+01 |
| VPS54 | Isoform of Q9P1Q0, Isoform 4 of Vacuolar protein sorting-associated protein 54 OS=Homo sapiens GN=VPS54 - [VPS54_HUMAN] | 1.28E+00 | 2.24E+00 |
| VPS8 | Isoform of Q8N3P4, Vacuolar protein sorting-associated protein 8 homolog (Fragment) OS=Homo sapiens GN=VPS8 PE=1 SV=1 - [C9JKL0_HUMAN] | 2.83E+00 | 3.14E+01 |
| VPS13B | Isoform of Q7Z7G8, Isoform 2 of Vacuolar protein sorting-associated protein 13B OS=Homo sapiens GN=VPS13B - [VP13B_HUMAN] | 2.56E+00 | 8.63E+00 |
| VPS13D | Isoform of Q5THJ4, Vacuolar protein sorting-associated protein 13D (Fragment) OS=Homo sapiens GN=VPS13D PE=1 SV=1 - [H3BLS7_HUMAN] | 1.91E+00 | 8.53E+00 |
| VPS18 | Vacuolar protein sorting-associated protein 18 homolog OS=Homo sapiens GN=VPS18 PE=1 SV=2 - [VPS18_HUMAN] | 2.78E+00 | 2.97E+01 |
| **Cytoskeleton Regulation** | | | |
| GSN | "Isoform of P06396, Gelsolin (Fragment) OS=Homo sapiens GN=GSN PE=1 SV=1 - [Q5T0I1_HUMAN];""Isoform of P06396, Isoform 2 of Gelsolin OS=Homo sapiens GN=GSN - [GELS_HUMAN]""" | 4.09E+00 | 1.33E+01 |
| MYLK3 | Isoform of Q32MK0, Isoform 2 of Myosin light chain kinase 3 OS=Homo sapiens GN=MYLK3 - [MYLK3_HUMAN] | 1.08E+00 | 1.29E+00 |
| ANXA3 | Isoform of P12429, Annexin (Fragment) OS=Homo sapiens GN=ANXA3 PE=1 SV=1 - [D6RFG5_HUMAN] | 6.28E+01 | 3.49E+02 |
| NSF | Isoform of P46459, Isoform 2 of Vesicle-fusing ATPase OS=Homo sapiens GN=NSF - [NSF_HUMAN] | 2.70E+00 | 2.68E+01 |
| OTOF | Isoform of Q9HC10, Isoform 2 of Otoferlin OS=Homo sapiens GN=OTOF - [OTOF_HUMAN] | 2.68E+02 | 7.79E+02 |
| ACTB | "Actin, cytoplasmic 1 OS=Homo sapiens GN=ACTB PE=1 SV=1 - [ACTB_HUMAN];""Actin, cytoplasmic 1 OS=Homo sapiens GN=ACTB PE=1 SV=1 - [ACTB_HUMAN]\|Beta-actin-like protein 2 OS=Homo sapiens GN=ACTBL2 PE=1 SV=2 - [ACTBL_HUMAN]""" | 9.07E+03 | 6.56E+02 |
| ACTG1 | Isoform of P63261, Actin, cytoplasmic 2 (Fragment) OS=Homo sapiens GN=ACTG1 PE=1 SV=1 - [I3L1U9_HUMAN] | 2.75E+00 | 2.85E+01 |
| ANXA2 | "Isoform of P07355, Annexin (Fragment) OS=Homo sapiens GN=ANXA2 PE=1 SV=1 - [H0YNA0_HUMAN];""Isoform of P07355, Annexin A2 (Fragment) OS=Homo sapiens GN=ANXA2 PE=1 SV=1 - [H0YMD9_HUMAN]"";""Isoform of P07355, Annexin (Fragment) OS=Homo sapiens GN=ANXA2 PE=1 SV=1 - [H0YKZ7_HUMAN]"";""Isoform of P07355, Annexin (Fragment) OS=Homo sapiens GN=ANXA2 PE=1 SV=1 - [H0YMD0_HUMAN]"";""Annexin A2 OS=Homo sapiens GN=ANXA2 PE=1 SV=2 - [ANXA2_HUMAN]"";""Isoform of P07355, Annexin A2 (Fragment) OS=Homo sapiens GN=ANXA2 PE=1 SV=1 - [H0YLE2_HUMAN]"";""Isoform of P07355, Annexin (Fragment) OS=Homo sapiens GN=ANXA2 PE=1 SV=1 - [H0YNP5_HUMAN]"";""Isoform of P07355, Annexin (Fragment) OS=Homo sapiens GN=ANXA2 PE=1 SV=1 - [H0YKS4_HUMAN]""" | 7.84E+03 | 1.41E+02 |
| ANXA6 | Isoform of P08133, Isoform 2 of Annexin A6 OS=Homo sapiens GN=ANXA6 - [ANXA6_HUMAN] | 2.29E+01 | 2.27E+02 |
| FLNA | "Isoform of P21333, Filamin-A OS=Homo sapiens GN=FLNA PE=1 SV=1 - [Q5HY54_HUMAN];""Isoform of P21333, Filamin-A (Fragment) OS=Homo sapiens GN=FLNA PE=1 SV=1 - [H0Y5C6_HUMAN]""" | 5.39E+00 | 2.09E+01 |
| MSN | Moesin OS=Homo sapiens GN=MSN PE=1 SV=3 - [MOES_HUMAN] | 2.04E+00 | 1.07E+01 |
| MYH9 | Isoform of P35579, Isoform 2 of Myosin-9 OS=Homo sapiens GN=MYH9 - [MYH9_HUMAN] | 3.22E+00 | 1.38E+01 |
| PFN1 | Profilin-1 OS=Homo sapiens GN=PFN1 PE=1 SV=2 - [PROF1_HUMAN] | 1.08E+00 | 1.29E+00 |
| TUBA1B | "Isoform of P68363, Tubulin alpha-1B chain (Fragment) OS=Homo sapiens GN=TUBA1B PE=4 SV=1 - [F8VRK0_HUMAN];""Isoform of P68363, Isoform 2 of Tubulin alpha-1B chain OS=Homo sapiens GN=TUBA1B - [TBA1B_HUMAN]""" | 1.55E+01 | 6.39E+01 |

| | | | |
|---|---|---|---|
| **Other Protein Cargo** | | | |
| ATP1A1 | Sodium/potassium-transporting ATPase subunit alpha-1 OS=Homo sapiens GN=ATP1A1 PE=1 SV=1 - [AT1A1_HUMAN] | 2.86E+00 | 3.28E+01 |
| CCT3 | Isoform of P49368, T-complex protein 1 subunit gamma OS=Homo sapiens GN=CCT3 PE=1 SV=1 - [B4DUR8_HUMAN] | 2.97E+00 | 3.70E+01 |
| CCT5 | Isoform of P48643, Isoform 2 of T-complex protein 1 subunit epsilon OS=Homo sapiens GN=CCT5 - [TCPE_HUMAN] | 2.96E+00 | 3.66E+01 |
| EEF1A1 | "Isoform of P68104, Elongation factor 1-alpha 1 OS=Homo sapiens GN=EEF1A1 PE=1 SV=1 - [A0A087WVQ9_HUMAN];""Isoform of P68104, Elongation factor 1-alpha OS=Homo sapiens GN=EEF1A1 PE=1 SV=1 - [A0A087WV01_HUMAN]""" | 2.99E+00 | 1.17E+01 |
| EEF2 | Elongation factor 2 OS=Homo sapiens GN=EEF2 PE=1 SV=4 - [EF2_HUMAN] | 8.25E+00 | 4.62E+01 |
| ENO1 | "Alpha-enolase OS=Homo sapiens GN=ENO1 PE=1 SV=2 - [ENOA_HUMAN];""Isoform of P06733, Alpha-enolase (Fragment) OS=Homo sapiens GN=ENO1 PE=1 SV=1 - [K7EM90_HUMAN]"";""Isoform of P06733, Isoform MBP-1 of Alpha-enolase OS=Homo sapiens GN=ENO1 - [ENOA_HUMAN]""" | 4.30E+01 | 2.21E+02 |
| GAPDH | "Isoform of P04406, Glyceraldehyde-3-phosphate dehydrogenase OS=Homo sapiens GN=GAPDH PE=1 SV=1 - [E7EUT5_HUMAN];""Isoform of P04406, Isoform 2 of Glyceraldehyde-3-phosphate dehydrogenase OS=Homo sapiens GN=GAPDH - [G3P_HUMAN]""" | 2.08E+02 | 3.11E+02 |
| HSP90AA1 | "Isoform of P07900, Isoform 2 of Heat shock protein HSP 90-alpha OS=Homo sapiens GN=HSP90AA1 - [HS90A_HUMAN]\|Heat shock protein HSP 90-beta OS=Homo sapiens GN=HSP90AB1 PE=1 SV=4 - [HS90B_HUMAN];""Isoform of P07900, Isoform 2 of Heat shock protein HSP 90-alpha OS=Homo sapiens GN=HSP90AA1 - [HS90A_HUMAN]""" | 1.59E+00 | 4.64E+00 |
| HSP90AB1 | Heat shock protein HSP 90-beta OS=Homo sapiens GN=HSP90AB1 PE=1 SV=4 - [HS90B_HUMAN] | 4.94E+00 | 1.93E+01 |
| HSPA8 | "Isoform of P11142, Heat shock cognate 71 kDa protein (Fragment) OS=Homo sapiens GN=HSPA8 PE=1 SV=1 - [E9PN25_HUMAN];""Isoform of P11142, Heat shock cognate 71 kDa protein (Fragment) OS=Homo sapiens GN=HSPA8 PE=1 SV=1 - [E9PI65_HUMAN]""" | 4.95E+00 | 3.89E+01 |
| LGALS3BP | Isoform of Q08380, Galectin-3-binding protein (Fragment) OS=Homo sapiens GN=LGALS3BP PE=1 SV=1 - [K7ESM3_HUMAN] | 3.46E+00 | 2.77E+01 |
| PGK1 | Isoform of P00558, Isoform 2 of Phosphoglycerate kinase 1 OS=Homo sapiens GN=PGK1 - [PGK1_HUMAN] | 1.90E+00 | 8.45E+00 |
| PKM | "Isoform of P14618, Pyruvate kinase OS=Homo sapiens GN=PKM PE=1 SV=1 - [H3BQ34_HUMAN];""Isoform of P14618, Pyruvate kinase OS=Homo sapiens GN=PKM PE=1 SV=1 - [B4DNK4_HUMAN]""" | 1.74E+01 | 1.31E+02 |
| PPIA | Isoform of P62937, Peptidyl-prolyl cis-trans isomerase OS=Homo sapiens GN=PPIA PE=1 SV=1 - [F8WE65_HUMAN] | 2.80E+00 | 2.39E+01 |
| PRDX2 | Peroxiredoxin-2 OS=Homo sapiens GN=PRDX2 PE=1 SV=5 - [PRDX2_HUMAN] | 2.71E+00 | 2.73E+01 |
| TPI1 | "Isoform of P60174, Isoform 4 of Triosephosphate isomerase OS=Homo sapiens GN=TPI1 - [TPIS_HUMAN];""Isoform of P60174, Triosephosphate isomerase (Fragment) OS=Homo sapiens GN=TPI1 PE=1 SV=1 - [U3KPZ0_HUMAN]""" | 2.96E+00 | 9.59E+00 |
| YWHAZ | Isoform of P63104, 14-3-3 protein zeta/delta (Fragment) OS=Homo sapiens GN=YWHAZ PE=1 SV=8 - [E5RGE1_HUMAN] | 1.33E+01 | 3.91E+01 |

**Supplementary Table 5.** PDAC cell line exosome proteins also identified by mass spectrometry analysis as bound to circulating immunoglobulins in the plasma of PDAC patients

| | Surface | | | | Surface Enrichment Ratio† | | Total Extract | | | | HLA-II (N. PDAC cell lines)‡ | |
|---|---|---|---|---|---|---|---|---|---|---|---|---|
| **Gene** | **Cell Mean MS2** | **Exosome Mean MS2** | **Ratio Exosome/Cell** | **P Value*** | **Cells** | **Exosomes** | **Cell Mean MS2** | **Exosome Mean MS2** | **Ratio Exosome/Cell** | **P Value*** | **$IC_{50}$<50 nM** | **$IC_{50}$<500 nM** |
| **ACTA1** | 2.8 | 358.5 | 129.57 | **0.0379** | 2.8 | 0.85 | NA | 421.8 | 421.78 | **0.0027** | | |
| **ACTB** | 193.9 | 266.9 | 1.38 | 0.2403 | 0.8 | 0.84 | 254.1 | 317.3 | 1.25 | 0.3768 | 5 | 3 |
| **ANXA1** | 187.6 | 107.3 | 0.57 | 0.0411 | 0.7 | 1.55 | 265.9 | 69.2 | 0.26 | 0.0152 | | |
| **ARF6** | 2.5 | 6.0 | 2.47 | 0.0921 | 0.3 | 1.04 | 8.9 | 5.8 | 0.66 | 0.2607 | | |
| **ATP5B** | 151.6 | 6.2 | 0.04 | 0.0050 | 1.2 | 1.36 | 128.1 | 4.6 | 0.04 | 0.0050 | | |
| **GSTP1** | 55.3 | 9.0 | 0.16 | 0.0022 | 0.2 | 0.81 | 225.1 | 11.1 | 0.05 | 0.0022 | 1 | 4 |
| **H2AFV/X** | NA | 141.3 | 141.27 | **0.0028** | 0.0 | 7.36 | NA | 19.2 | 19.20 | **0.0028** | | |
| **HSPA8** | NA | 749.6 | 749.55 | **0.0027** | 0.0 | 1.03 | NA | 726.3 | 726.34 | **0.0027** | | 4 |
| **JUP** | 43.8 | 36.8 | 0.84 | 0.5887 | 5.3 | 1.81 | 8.2 | 20.4 | 2.47 | 0.2946 | | |
| **KRT10** | 116.7 | 36.2 | 0.31 | 0.0260 | 5.4 | 6.10 | 21.8 | 5.9 | 0.27 | 0.1727 | 3 | 3 |
| **KRT16** | 12.2 | 8.4 | 0.69 | 0.8089 | 3.9 | 1.86 | 3.1 | 4.5 | 1.44 | 1.0000 | | |
| **KRT17** | 2.4 | 26.6 | 11.23 | **0.0124** | 1.2 | 5.60 | 2.0 | 4.8 | 2.37 | 0.1424 | | |
| **KRT20** | NA | 5.7 | 5.67 | **0.0096** | 0.8 | 2.46 | 1.3 | 2.3 | 1.84 | 0.2530 | | 1 |
| **KRT5** | 4.5 | 9.6 | 2.10 | 0.0649 | 3.0 | 4.22 | 1.5 | 2.3 | 1.47 | 0.5611 | 1 | |
| **LGALS3BP** | 110.7 | 600.0 | 5.42 | **0.0022** | 0.7 | 1.20 | 168.5 | 500.6 | 2.97 | **0.0260** | | |
| **NAGK** | 1.7 | 1.8 | 1.03 | 0.7976 | 0.4 | 0.27 | 4.5 | 6.5 | 1.44 | 0.6991 | | |
| **NME2P1** | 2.0 | 19.7 | 9.78 | 0.0562 | 0.1 | 4.62 | 34.3 | 4.3 | 0.12 | 0.0538 | | |
| **PABPC4** | 1.5 | 8.9 | 5.78 | 0.0608 | 1.5 | 0.86 | NA | 10.4 | 10.37 | **0.0096** | | |
| **PCBP1** | 4.7 | 9.8 | 2.07 | 0.0931 | 0.4 | 0.95 | 12.7 | 10.3 | 0.81 | 0.5887 | 1 | 1 |
| **PDE4DIP** | NA | 2.7 | 2.67 | 0.1757 | 0.9 | 0.53 | 1.1 | 5.1 | 4.61 | 0.3261 | | |
| **PKM/PKM2** | 637.9 | 629.2 | 0.99 | 1.0000 | 1.0 | 0.60 | 582.4 | 1053.2 | 1.81 | **0.0260** | | 1 |
| **POTEE** | NA | 107.8 | 107.83 | **0.0028** | 0.0 | 1.11 | NA | 97.5 | 97.51 | **0.0028** | | |
| **POTEF** | 1.3 | 98.5 | 75.66 | **0.0048** | 1.3 | 1.42 | NA | 69.4 | 69.39 | **0.0027** | | |
| **PSMA7** | 1.4 | 201.5 | 142.62 | **0.0037** | 0.1 | 4.75 | 20.1 | 42.4 | 2.10 | 0.0776 | | |
| **RAN** | 3.4 | 46.9 | 13.93 | **0.0278** | 3.4 | 0.46 | NA | 101.1 | 101.10 | **0.0028** | | |
| **RPSA** | 4.5 | 72.7 | 16.05 | **0.0022** | 0.1 | 1.21 | 36.0 | 60.0 | 1.67 | **0.0152** | | |
| **TUBB** | 102.2 | 581.1 | 5.68 | **0.0050** | 1.4 | 1.00 | 75.0 | 578.8 | 7.72 | **0.0050** | | 1 |
| **TUBB2A** | 1.2 | 16.3 | 14.03 | 0.1044 | 0.7 | 0.91 | 1.7 | 17.9 | 10.48 | 0.1044 | | |
| **TUBB2B** | NA | 148.2 | 148.21 | **0.0028** | 0.0 | 0.93 | NA | 159.8 | 159.79 | **0.0028** | | |
| **TUBB3** | 2.3 | 308.5 | 136.25 | **0.0022** | 0.9 | 0.99 | 2.6 | 312.3 | 121.17 | **0.0050** | | |
| **TUBB4A** | 1.9 | 269.0 | 145.26 | **0.0050** | 1.7 | 1.15 | 1.1 | 233.2 | 217.33 | **0.0037** | | |
| **TUBB4B** | 23.3 | 178.0 | 7.64 | **0.0022** | 1.0 | 1.01 | 23.6 | 176.0 | 7.46 | **0.0022** | | |
| **UBA52** | NA | 98.1 | 98.10 | **0.0028** | 0.0 | 2.10 | NA | 46.7 | 46.65 | **0.0028** | 1 | |
| **UBC** | NA | 70.0 | 70.03 | **0.0028** | 0.0 | 2.50 | NA | 28.0 | 28.01 | **0.0028** | | |

*NA* indicates protein not quantified

*P value was calculated by Mann-Whitney t-test. Bold indicates proteins showing significantly higher expression levels in exosome compared to cell extracts.

† Surface enrichment indicates the ratio of surface to total extract MS2 counts.

‡ Number of PDAC cell lines (out of 6) in which peptides for this protein were identified with the specified $IC_{50}$.

**Supplementary Table 6.** Patient characteristics of PDAC plasma samples applied for autoantibody analyses

| | | Set #1 | | | Set #2 | | Set #3 | | |
|---|---|---|---|---|---|---|---|---|---|
| | | Pancreatic cancer | Healthy controls | Chronic pancreatitis | Pancreatic cancer | Healthy controls | Pancreatic cancer | Healthy controls | Chronic pancreatitis |
| **Total (*n*)** | | 10 | 10 | 10 | 13 | 13 | 42 | 50 | 50 |
| **Gender (*n*)** | **Male** | 4 | 4 | 6 | 11 | 11 | 26 | 31 | 31 |
| | **Female** | 6 | 6 | 4 | 2 | 2 | 16 | 19 | 19 |
| **Age (mean (s.d.))** | | 74.2 (8.6) | 60.2 (10.4) | 61.6 (13.3) | 68.3 (4.1) | 68.1 (4.2) | 64.4 (12.0) | 68.6 (8.3) | 57.9 (14.2) |
| **Stage (*n*)** | **IA** | - | - | - | 1 | - | 3 | - | - |
| | **IB** | 2 | - | - | 2 | - | 9 | - | - |
| | **IIA** | 1 | - | - | - | - | 30 | - | - |
| | **IIB** | 7 | - | - | 3 | - | - | - | - |
| | **III** | - | - | - | 2 | - | - | - | - |
| | **IV** | - | - | - | 3 | - | - | - | - |
| | **Resectable (No TNM data)** | - | - | - | - | - | - | - | - |
| | **ND** | - | - | - | 2 | - | - | - | - |
| **Timing of sample collection** | | At time of diagnosis | | | 9.9 (in average; range 7-13) months prior to diagnosis | | At time of diagnosis | | |
| **Analysis** | **Figure** | | | | | | | | |
| *Western blot* | *Supp. Fig. 9* | x | | | | | | | |
| *Protein arrays* | *Table 1* | x | | | x | | x | | |
| *TEM* | *Fig. 6a* | x | | | | | | | |
| *Luminex* | *Fig. 6b* | | | | x | | | | |
| *CDC* | *Fig. 6c & d* | | | | x | | | | |

**Supplementary Table 7.** Exosome proteins identified by mass spectrometry in bands immunoreactive against PDAC patient plasmas.

| | | | Surface | | | | Surface Enrichment Ratio[¶] | | Total Extract | | | |
|---|---|---|---|---|---|---|---|---|---|---|---|---|
| **Gene** | **PDAC Pool*** | **Band N.†** | **Cells Mean MS2** | **Exosome Mean MS2** | **Ratio Exosome/Cell** | **P Value‡** | **Cells** | **Exosomes** | **Cell Mean MS2** | **Exosome Mean MS2** | **Ratio Exosome/Cell** | **P Value‡** |
| **ACTA1** | Early | 3 | 2.8 | 358.5 | 129.57 | **0.0379** | 2.77 | 0.85 | NA | 421.8 | 421.78 | **0.0027** |
| **ACTB** | Early | 3 | 193.9 | 266.9 | 1.38 | 0.2403 | 0.76 | 0.84 | 254.1 | 317.3 | 1.25 | 0.3768 |
| **ACTR3** | Early | 2 | NA | 11.4 | 11.43 | **0.0096** | NA | 0.21 | NA | 54.5 | 54.48 | **0.0028** |
| **AIMP1** | Early-Adv | 4 | 17.6 | 7.1 | 0.41 | 0.3776 | 2.38 | 2.23 | 7.4 | 3.2 | 0.43 | 0.1255 |
| **ALDH1A1** | Early-Adv | 1 | 5.5 | 2.4 | 0.43 | 0.5320 | 0.41 | 0.53 | 13.6 | 4.5 | 0.33 | 0.4550 |
| **ALDH1A3** | Early-Adv | 1 | 56.7 | 4.3 | 0.08 | 0.0194 | 1.13 | 0.18 | 50.1 | 23.3 | 0.47 | 0.2971 |
| **ALDOA** | Early | 3 | 154.6 | 97.7 | 0.63 | 0.4225 | 0.29 | 0.98 | 538.7 | 99.7 | 0.19 | 0.0050 |
| **ANXA1** | Early-Adv | 4 | 187.6 | 107.3 | 0.57 | 0.0411 | 0.71 | 1.55 | 265.9 | 69.2 | 0.26 | 0.0152 |
| **ANXA2** | Early-Adv | 4 | NA | 1050.3 | 1050.35 | **0.0026** | 0.43 | 1.06 | 2.3 | 986.7 | 422.21 | **0.0034** |
| **APOE** | Early-Adv | 4 | 2.7 | 33.2 | 12.22 | 0.1031 | 1.15 | 4.90 | 2.4 | 6.8 | 2.87 | 0.3472 |
| **C22orf28** | Early-Adv | 1 | 2.5 | 5.1 | 2.02 | 0.5497 | 2.55 | 0.80 | NA | 6.4 | 6.42 | **0.0096** |
| **CAP1** | Early-Adv | 1 | 104.0 | 24.1 | 0.23 | 0.0022 | 0.86 | 1.93 | 121.0 | 12.5 | 0.10 | 0.0022 |
| **CCT2** | Early-Adv | 1 | 102.1 | 114.8 | 1.13 | 0.1320 | 0.62 | 0.51 | 165.2 | 226.7 | 1.37 | 0.3095 |
| **CCT4** | Early-Adv | 1 | 54.1 | 48.2 | 0.89 | 0.3939 | 1.66 | 0.46 | 32.6 | 105.9 | 3.25 | **0.0022** |
| **CCT6A** | Early-Adv | 1 | 40.5 | 15.9 | 0.39 | 0.3095 | 0.75 | 0.33 | 54.3 | 48.8 | 0.90 | 0.8182 |
| **DARS** | Early-Adv | 1 | 39.3 | 8.4 | 0.22 | 0.0450 | 2.18 | 0.12 | 18.0 | 68.3 | 3.79 | **0.0022** |
| **DDX39B** | Early | 2 | NA | 34.7 | 34.73 | **0.0028** | NA | 0.40 | NA | 87.0 | 86.97 | **0.0028** |
| **DDX6** | Early-Adv | 1 | 18.3 | 4.9 | 0.27 | 0.2290 | 1.01 | 0.62 | 18.0 | 7.9 | 0.44 | 0.4848 |
| **EEF1A1** | Early | 2 | NA | 67.7 | 67.65 | **0.0028** | NA | 0.55 | NA | 123.5 | 123.50 | **0.0028** |
| **EEF1A2** | Early | 2 | 12.2 | 37.3 | 3.06 | 0.0931 | 0.67 | 0.67 | 18.3 | 55.7 | 3.05 | 0.4848 |
| **EEF1G** | Early-Adv | 1,2 | 103.4 | 64.2 | 0.62 | 0.0043 | 0.87 | 0.61 | 118.4 | 106.0 | 0.89 | 0.5887 |
| **EIF2S1** | Early-Adv | 4 | 8.8 | 7.0 | 0.80 | 0.9372 | 0.93 | 0.37 | 9.5 | 19.0 | 2.00 | 0.1797 |
| **EIF2S3** | Early-Adv | 1,2 | 3.8 | 3.9 | 1.03 | 1.0000 | 0.64 | 0.12 | 5.8 | 31.6 | 5.42 | **0.0022** |
| **EIF3I** | Early-Adv | 4 | 11.5 | 14.2 | 1.23 | 0.6991 | 0.64 | 0.73 | 18.1 | 19.4 | 1.07 | 1.0000 |
| **EIF3M** | Early | 3 | NA | 29.3 | 29.27 | **0.0028** | NA | 2.16 | NA | 13.5 | 13.52 | **0.0096** |
| **ENO1** | Early | 2 | 616.4 | 83.9 | 0.14 | 0.0050 | 0.53 | 0.80 | 1164.7 | 105.1 | 0.09 | 0.0049 |
| **GAPDH** | Early-Adv | 4 | 645.1 | 109.8 | 0.17 | 0.0050 | 0.82 | 0.51 | 789.1 | 214.1 | 0.27 | 0.0049 |
| **GNAI3** | Early | 3 | 3.3 | 20.9 | 6.34 | **0.0087** | 2.91 | 1.77 | 1.1 | 11.8 | 10.42 | **0.0250** |
| **GNB2L1** | Advanced | 5 | 1.0 | 189.6 | 181.68 | **0.0037** | 1.04 | 0.32 | NA | 590.9 | 590.87 | **0.0028** |
| **H2AFX** | Early | 7 | 1.5 | 17.4 | 11.51 | **0.0438** | 0.74 | 17.45 | 2.0 | NA | 0.49 | 0.0740 |
| **HIST2H2AC** | Early | 7 | NA | 22.4 | 22.43 | **0.0096** | NA | 6.50 | NA | 3.5 | 3.45 | 0.0740 |
| **HLA-A** | Early | 3 | 278.5 | 713.0 | 2.56 | **0.0303** | 7.78 | 2.44 | 35.8 | 292.0 | 8.16 | **0.0043** |
| **HSPA8** | Early-Adv | 1 | NA | 749.6 | 749.55 | **0.0027** | NA | 1.03 | NA | 726.3 | 726.34 | **0.0027** |
| **HSPB1** | Early | 6 | 65.8 | 51.5 | 0.78 | 0.6991 | 1.22 | 2.86 | 53.9 | 18.0 | 0.33 | 0.1797 |
| **IMPDH2** | Early-Adv | 1 | 14.2 | 29.0 | 2.04 | **0.0043** | 0.32 | 1.61 | 44.3 | 17.9 | 0.40 | 0.1320 |
| **KPNA2** | Early-Adv | 1 | 6.5 | 29.9 | 4.61 | **0.0022** | 1.80 | 2.58 | 3.6 | 11.6 | 3.22 | 0.1320 |
| **KRT7** | Early-Adv | 1 | 21.3 | 24.0 | 1.13 | 0.8182 | 1.11 | 2.04 | 19.2 | 11.8 | 0.61 | 0.5683 |
| **KRT8** | Early-Adv | 1 | 125.1 | 297.5 | 2.38 | **0.0152** | 0.61 | 1.30 | 205.0 | 229.0 | 1.12 | 0.6884 |
| **MMP7** | Early | 6 | 2.7 | 7.9 | 2.94 | 0.5718 | 0.61 | 1.59 | 4.4 | 5.0 | 1.12 | 0.9338 |
| **NAMPT** | Early-Adv | 1 | 53.2 | NA | 0.02 | 0.0028 | 3.21 | 0.21 | 16.6 | 4.8 | 0.29 | 0.0411 |
| **PGAM1** | Early | 6 | 51.1 | 19.0 | 0.37 | 0.0043 | 0.55 | 2.17 | 92.9 | 8.8 | 0.09 | 0.0022 |
| **PKM/PKM2** | Early-Adv | 1 | 637.9 | 629.2 | 0.99 | 1.0000 | 1.04 | 0.60 | 582.4 | 1053.2 | 1.81 | **0.0260** |
| **PPP1CA** | Early-Adv | 4 | 4.6 | 3.1 | 0.67 | 0.4942 | 0.45 | 0.06 | 10.3 | 48.5 | 4.72 | **0.0303** |
| **PPP1CB** | Early-Adv | 4 | NA | 4.4 | 4.40 | **0.0284** | NA | 0.17 | NA | 25.7 | 25.72 | **0.0028** |
| **PPP2CA** | Early-Adv | 4 | NA | 7.5 | 7.47 | **0.0096** | NA | 0.36 | NA | 20.9 | 20.92 | **0.0028** |
| **PRMT1** | Early | 3 | 16.3 | 8.6 | 0.53 | 0.4848 | 2.62 | 0.47 | 6.2 | 18.3 | 2.94 | 0.1994 |
| **PRPF19** | Early-Adv | 1 | 1.4 | 3.4 | 2.35 | 0.1805 | 1.11 | 0.31 | 1.3 | 11.0 | 8.46 | **0.0043** |
| **PSMA1** | Advanced | 5 | 6.9 | 144.6 | 20.93 | **0.0022** | 0.35 | 2.75 | 19.7 | 52.5 | 2.66 | 0.0649 |
| **PSMA5** | Early | 6 | 6.7 | 67.4 | 10.06 | **0.0022** | 0.09 | 1.71 | 71.7 | 39.5 | 0.55 | 0.0043 |
| **PSMA6** | Early | 6 | 2.2 | 144.0 | 64.89 | **0.0022** | 0.36 | 3.93 | 6.1 | 36.7 | 5.99 | **0.0438** |
| **PSMA7** | Early | 6 | 1.4 | 201.5 | 142.62 | **0.0037** | 0.07 | 4.75 | 20.1 | 42.4 | 2.10 | 0.0776 |
| **PSMC2** | Early | 2 | 5.1 | 48.8 | 9.60 | **0.0049** | 0.47 | 0.61 | 10.8 | 79.8 | 7.42 | **0.0022** |
| **PSMC3** | Early | 2 | 9.2 | 85.4 | 9.29 | **0.0022** | 0.54 | 0.92 | 17.1 | 92.5 | 5.42 | **0.0260** |
| **PSMC4** | Early | 2 | 8.6 | 26.3 | 3.04 | **0.0022** | 0.57 | 0.62 | 15.1 | 42.4 | 2.81 | 0.0649 |
| **PSMD12** | Early-Adv | 1 | 6.5 | 21.5 | 3.30 | **0.0087** | 2.09 | 0.47 | 3.1 | 46.1 | 14.79 | **0.0022** |
| **PSMD14** | Early-Adv | 4 | 3.4 | 16.4 | 4.82 | **0.0050** | 0.56 | 1.54 | 6.1 | 10.6 | 1.75 | 0.0649 |
| **PSMD7** | Early | 3 | 1.4 | 62.4 | 45.35 | **0.0022** | 0.55 | 1.69 | 2.5 | 37.0 | 14.84 | **0.0022** |
| **RAB5B** | Early | 6 | 2.2 | 22.4 | 10.19 | **0.0081** | 0.50 | 2.80 | 4.4 | 8.0 | 1.80 | 0.2615 |
| **RAB5C** | Early | 6 | 14.7 | 43.2 | 2.94 | **0.0043** | 0.95 | 2.77 | 15.5 | 15.6 | 1.01 | 0.4848 |
| **RAN** | Early | 6 | 3.4 | 46.9 | 13.93 | **0.0278** | 3.37 | 0.46 | NA | 101.1 | 101.10 | **0.0028** |
| **RPL7** | Early | 6 | 3.0 | 2.1 | 0.70 | 0.2894 | 0.29 | 0.50 | 10.6 | 4.2 | 0.40 | 0.1727 |
| **RPL7A** | Early | 6 | 38.0 | 3.8 | 0.10 | 0.0050 | 2.44 | 0.57 | 15.6 | 6.7 | 0.43 | 0.2403 |
| **RPLP0** | Early-Adv | 4 | 6.3 | 64.8 | 10.28 | **0.0194** | 1.88 | 0.50 | 3.4 | 129.7 | 38.67 | **0.0072** |
| **RPS4X** | Early | 6 | 3.1 | 3.9 | 1.27 | 0.7976 | 0.94 | 0.50 | 3.3 | 7.9 | 2.38 | 0.4550 |
| **RPSA** | Early | 6 | 4.5 | 72.7 | 16.05 | **0.0022** | 0.13 | 1.21 | 36.0 | 60.0 | 1.67 | **0.0152** |
| **RUVBL1** | Early-Adv | 1 | 8.1 | 81.7 | 10.13 | **0.0022** | 0.74 | 0.87 | 10.8 | 93.8 | 8.64 | **0.0043** |
| **RUVBL2** | Early-Adv | 1,2 | 10.6 | 86.5 | 8.20 | **0.0022** | 2.28 | 0.80 | 4.6 | 108.2 | 23.42 | **0.0048** |
| **SDCBP** | Advanced | 5 | 1.3 | 118.3 | 94.39 | **0.0049** | 0.73 | 1.23 | 1.7 | 96.5 | 56.47 | **0.0049** |
| **STRAP** | Early | 3 | 1.5 | 7.1 | 4.87 | 0.0608 | 0.15 | 0.26 | 9.9 | 27.2 | 2.75 | 0.4704 |
| **TALDO1** | Early-Adv | 4 | 47.5 | 8.4 | 0.18 | 0.0152 | 0.52 | 1.12 | 91.6 | 7.5 | 0.08 | 0.0043 |
| **TUBA1A** | Early-Adv | 1,2 | NA | 239.1 | 239.14 | **0.0028** | NA | 1.13 | NA | 211.9 | 211.87 | **0.0028** |
| **TUBA1B** | Early-Adv | 1,2 | NA | 238.5 | 238.55 | **0.0027** | NA | 1.18 | NA | 202.5 | 202.49 | **0.0027** |
| **TUBA1C** | Early-Adv | 1,2 | NA | 160.5 | 160.51 | **0.0028** | NA | 0.94 | NA | 171.4 | 171.38 | **0.0028** |
| **TUBB** | Early-Adv | 1,2 | 102.2 | 581.1 | 5.68 | **0.0050** | 1.36 | 1.00 | 75.0 | 578.8 | 7.72 | 0.0050 |
| **TUBB2B** | Early-Adv | 1,2 | NA | 148.2 | 148.21 | **0.0028** | NA | 0.93 | NA | 159.8 | 159.79 | **0.0028** |
| **TUBB3** | Early-Adv | 1,2 | 2.3 | 308.5 | 136.25 | **0.0022** | 0.88 | 0.99 | 2.6 | 312.3 | 121.17 | **0.0050** |
| **TUBB4A** | Early-Adv | 1,2 | 1.9 | 269.0 | 145.26 | **0.0050** | 1.73 | 1.15 | 1.1 | 233.2 | 217.33 | **0.0037** |
| **TUBB4B** | Early-Adv | 1,2 | 23.3 | 178.0 | 7.64 | **0.0022** | 0.99 | 1.01 | 23.6 | 176.0 | 7.46 | **0.0022** |
| **TUBB6** | Early-Adv | 1,2 | 7.0 | 122.4 | 17.40 | **0.0022** | 1.40 | 0.85 | 5.0 | 143.9 | 28.70 | **0.0022** |
| **UGDH** | Early-Adv | 1 | 51.8 | 17.1 | 0.33 | 0.1320 | 0.78 | 0.41 | 66.0 | 41.2 | 0.62 | 0.3095 |
| **VIM** | Early-Adv | 1 | 26.7 | 190.5 | 7.14 | 0.0649 | 0.92 | 3.23 | 29.1 | 58.9 | 2.03 | 0.7483 |
| **YWHAQ** | Early | 6 | 14.5 | 46.5 | 3.21 | **0.0022** | 0.25 | 1.29 | 59.0 | 36.1 | 0.61 | 0.1320 |
| **YWHAZ** | Early | 6 | NA | 124.3 | 124.30 | **0.0028** | NA | 1.23 | NA | 101.3 | 101.31 | **0.0028** |

NA indicates protein not quantified

* PDAC pool showing immunoreactivity against the band containing this protein. Adv, advanced stage PDAC pool.

† Band number, as per Supplementary Figure 7, in which this protein was identified.

‡ P value was calculated by Mann-Whitney t-test. Bold indicates proteins showing significantly higher expression levels in exosome compared to cell extracts.

¶ Surface enrichment indicates the ratio of surface to total extract MS2 counts.
