# Supplementary material for: Exosomes harbor B cell targets in pancreatic adenocarcinoma and exert decoy function against complement-mediated cytotoxicity
Source: Nat Commun. 2019 Jan 16;10:254. doi: 10.1038/s41467-018-08109-6 (PMC6335434; doi:10.1038/s41467-018-08109-6)
Supplement: Supplementary file 5 — Description of Additional Supplementary Files [file 41467_2018_8109_MOESM5_ESM.docx]

**Title: Supplementary Data 1.**
**Description:** Proteins identified by mass spectrometry analysis in the Ig-bound fraction of PDAC patients and matched control plasma

**Title: Supplementary Data 2.
Description:** Sequence coverage of PDAC cell line exosome surfaceome proteins also identified by mass spectrometry analysis as bound to circulating immunoglobulins in the plasma of PDAC patients
